# Effect of emotion on hippocampal-dependent associative binding through the lens of the weather prediction task

**DOI:** 10.3758/s13415-025-01371-4

**Published:** 2025-12-10

**Authors:** Emilie de Montpellier, Hannah Bernhard, Richard Henson, Thomas G. W. McConnell, Deborah Talmi

**Affiliations:** 1 Department of Psychology, Downing Site, University of Cambridge, Cambridge, CB2 3EB UK; 2 MRC Cognition & Brain Sciences Unit, Department of Psychiatry, University of Cambridge, Cambridge, UK

**Keywords:** Associative memory, Statistical learning, Weather prediction task, Emotion, Paired-associates

## Abstract

We investigated the impact of negative emotion on hippocampal-dependent associative memory through the "weather prediction task," which distinguishes between hippocampal (declarative) and striatal (procedural) memory systems. This was achieved by comparing the “paired-associates” condition, where participants memorise associations between cues and outcomes, with the “feedback” condition, where they learn these associations through trial-and-error. Based on the dual representation theory, we hypothesized that negative emotion would selectively impair hippocampal-dependent memory but instead found substantial evidence for a null effect of emotion. Across three experiments, the third of which employed a sequential design with Bayesian statistics and a sample of 800 participants, negative emotion did not decrease associative memory accuracy in the hippocampal-dependent “paired-associates” condition. These results challenge the dual representation theory, at least in the context of nontraumatic, controlled laboratory conditions.

The dual representation theory (DRT) claims that when experiencing a traumatic situation, amygdala activity is enhanced, leading to a strong encoding of the sensory and affective components of the event, but weakened hippocampal-dependent associative memory (Bisby & Burgess, 2017; Brewin et al., 2010; Jacobs & Nadel, 1985; 1998). Associative memory is crucial for binding together the contextual aspects of an event into a coherent, episodic memory (Cohen & Eichenbaum, 1993; Davachi, 2006; Eichenbaum et al., 2007). According to DRT, poor hippocampal-dependent associative memory for the trauma is one of the main causes of intrusive memory in posttraumatic stress disorder (PTSD) (Brewin et al., 2010). While the DRT was developed to account for clinical cases of PTSD (Brewin et al., 2010; Brewin, 2014; Brewin & Field, 2024), Brewin and colleagues have used emotional film and picture stimuli with healthy samples in the laboratory to investigate the mechanisms that go awry in PTSD, claiming strong support for DRT from such studies in their 2020 review (Bisby et al., 2020). The core of this support is evidence that both signalling in the hippocampus and hippocampal-dependent associative memory are decreased for emotional stimuli (Bisby & Burgess, 2013; 2017; Bisby et al., 2016; 2018; Madan et al., 2012; 2017).

This work has had a substantial impact on the recent decade of research in experimental psychology, inspiring an emerging consensus that negative emotional arousal decreases associative memory (Palombo et al., 2021; Wang & Lapate, 2024; Berkers et al., 2016). Nevertheless, research on the impact of emotion on associative memory has resulted in inconsistent findings. Alongside findings that negative emotion decreases associative memory for unrelated items (Okada et al., 2011; Onoda et al., 2009), others report increases or null effects (Henson et al., 2016; Luck et al., 2014; Madan et al., 2020; Mickley Steinmetz et al., 2016; Sharot & Yonelinas, 2008). The DRT can explain this inconsistency by referring to the nature of the associations the memory test is sensitive to. Associative memories vary in the degree to which their informational elements come together within the medial temporal lobe (Mayes et al., 2007), and the DRT only predicts emotion-based decreases for hippocampal-dependent associations.

In three experiments, we aimed to obtain direct evidence for a selective causal influence of emotion on hippocampal-based associative memory by contrasting two similar conditions that depend on hippocampal binding to varying degrees. For this purpose, we employed the weather prediction task (WPT) with its two learning versions: Feedback (FB) and Paired-Associate (PA). It is generally agreed that the FB version is less hippocampal-dependent and more dependent on the striatal system (Gluck et al., 2002; Holl et al., 2012; Knowlton et al., 1996; Poldrack et al., 2001), whereas the PA version relies more on the hippocampus (Li et al., 2016; Poldrack et al., 2001; Shohamy, Myers, Grossman et al., 2004). Because the two versions of the WPT are extremely well-matched, the comparison between them offers the possibility to isolate the contribution of hippocampal-dependent processes.

## The Weather Prediction Task

Hippocampal-dependent associative memory, a type of declarative memory, is distinct from striatal-based memory, a type of nondeclarative, procedural memory (Cohen & Squire, 1980). The weather prediction task (WPT) offers a behavioral assay that is differentially sensitive to their contribution.

In the WPT, people learn to predict a binary outcome based on cues comprising of four images, traditionally tarot cards. Each card is associated with an outcome (“rain” or “shine”) with a different probability, and the combined probabilities determine the probability of the outcome for each set of cards. In the FB version of the WPT, participants learn to predict the outcome by receiving corrective feedback after prediction. Hence, participants learn gradually the cue-outcome associations and become better at predicting throughout the task (Gluck et al., 2002; Poldrack et al., 2001). This version of the task resembles habit learning (Packard & Knowlton, 2002)—a form of procedural memory that is not impaired in people with medial temporal lobe damage (Cavaco, 2004; Filoteo et al., 2001). Parkinson’s and Huntington’s patients, whose striatum is impacted by neurodegeneration, show decreased performance on the FB version of the WPT (Holl et al., 2012; Knowlton et al., 1996; Shohamy, Myers, Grossman et al., 2004). By contrast, amnesic patients presented no deficits, at least during the first 50 trials (Knowlton et al., 1996), even though they were unable to recall details about the testing session or recognize the visual stimuli presented during the task on a post-test questionnaire (see also Foerde et al., 2006). The PA version of the WPT does not use trial-by-trial feedback. Instead, the cue and the associated outcome are presented simultaneously to participants, who are simply asked to memorise the associations. This version of the WPT requires an ability to store novel associations between unrelated stimuli, a hallmark impairment in amnesia following hippocampal damage (Cohen & Eichenbaum, 1993; Eichenbaum et al., 2007).

Neuroimaging data support this neuropsychological double dissociation between the two WPT task versions. An influential early neuroimaging study showed that when comparing the two versions of the task with healthy volunteers, during the FB version activity in the caudate nucleus was increased, and activity in the MTL was reduced, whereas the opposite pattern was found in the PA version (Poldrack et al., 2001). Furthermore, greater striatal activation has been found to be related to increased FB task performance (Prince et al., 2012; Schwabe & Wolf, 2012). Aligning with these findings, a recent study demonstrated that performance on a FB version of the WPT was not impacted by hippocampal-network-targeted neurostimulation, despite this procedure increasing hippocampal connectivity with cortical regions, and correspondingly enhancing paired-associates learning (Freedberg et al., 2022). Taken together, these findings support the argument that compared to the FB version, the PA version of the WPT relies preferentially on hippocampal-dependent associative binding, although this does not mean the two versions are “process-pure” (Freedberg et al., 2022; Lagnado et al., 2006; Newell et al., 2007).

Optimal responding in the test stage of the WPT (also termed “accurate” responding) is defined as choosing the outcome that was associated most often with that set of cues during learning. When the cue includes more than one card, participants can observe a simple rule—focus on one card to predict the outcome—or integrate the information provided by the combination of all of the cards that make up the cue. These two strategies are termed “simple” and “complex,” respectively (Gluck et al., 2002). It is established that patients with Parkinson’s disease are less likely to use the optimal, complex strategies in the FB version (Shohamy, Myers, Onlaor et al., 2004), suggesting that their deployment depends on striatal function. This conclusion is supported by a negative correlation observed between simple (compared with complex) strategy deployment and striatal BOLD signal in participants who took part in the FB version (Schwabe & Wolf, 2012). Schwabe and Wolf also observed a positive correlation between simple (compared to complex) strategy deployment and BOLD signal in the hippocampus. They argued that participants who use simple strategies are more likely to rely on hippocampal-dependent, declarative memory to solve the task. However, recent research employing a neural network model of the hippocampal system indicated that the monosynaptic pathway between the entorhinal cortex and hippocampal subfield CA1 can support the ability to detect the regularities that define category structure across exemplars (Sučević & Schapiro, 2023). These results suggest that in some situations, the ability to deploy complex strategies may also rely on the hippocampus, potentially explaining why patients with hippocampal damage were unable to do so in a modified version of the FB-WPT, and therefore, performed poorly (Hopkins et al., 2004).

Previous studies suggest that negative emotional arousal and stress influence the strategies used by participants in the FB version. Prince and colleagues (2012) used a variant of the FB-WPT with either neutral or negative outcomes (flowers and mushrooms for neutral, or snakes and spiders for emotional, instead of the traditional “rain” and “shine” cartoon). They reported that the group with an emotional outcome was more likely to use complex strategies compared with the neutral group, suggesting that emotion promotes striatal-based strategies. The finding that participants in the emotional group used more complex strategies replicated an earlier finding that used similar stimuli, but only during the second day of learning (Thomas & LaBar, 2008). Accuracy among these groups and a group of more fearful participants was equivalent. Other studies revealed an initial impairment for emotional conditions of the FB (Steidl et al., 2006; 2011), which had disappeared by the end of the task. Schwabe and Wolf (2012) investigated the impact of stress on the FB version using the “cold pressor” test—a procedure that reliably induces a neuroendocrine stress response—administered before the WPT. The stress group tended to use more complex strategies compared with the neutral group, with no impact on overall task accuracy. Additionally, concurrent fMRI data indicated that striatal activity was correlated with performance in the stress group, whereas for the control group, hippocampal activity correlated with performance. Given their behavioural and fMRI data, the authors concluded that stress appears to favour the procedural system, in agreement with previous studies (Kim et al., 2001; Packard & Goodman, 2012; Schwabe et al., 2007; Wirz et al., 2018; see also Zerbes & Schwabe, 2021). Overall, these studies suggest that emotion influences the strategies used by participants in the FB version, such that negative emotional arousal and stress decrease the involvement of the hippocampus and facilitate reliance on a procedural, hippocampal-independent system during learning, a conclusion that aligns well with the DRT. However, the sole focus on the FB version of the task, where nonhippocampal systems can support associative binding, is a limitation of the studies that analysed strategy use. The DRT can be further supported by showing that negative emotional arousal decreases performance more in the PA version, where the contribution of hippocampal-dependent associative binding is greater.

## Present study

Our aim was to reveal, for the first time, evidence for a selective effect of negative emotional arousal on hippocampal-dependent associative memory. To achieve this aim, we contrasted conditions that rely preferentially on hippocampal- and striatal-dependent learning, the PA and FB versions of the WPT. The two tasks were matched in terms of the procedure, number of trials and stimuli used, so that only the presence of feedback could explain performance differences. To manipulate emotion, we made a small number of superficial changes to the WPT that did not alter its crucial conceptual logic. The most conspicuous change was replacing the tarot card images with natural scenes that were either negative and arousing or neutral, noting that the rationale of the WPT should not be affected by the visual properties of cue images. We derived our key hypothesis from the DRT, predicting that negative emotional arousal will impair performance in the PA condition more than in the FB condition.

The paradigm was a 2 (Task Version: FB vs. PA) x 2 (emotion: Neutral vs. Negative) factorial design. We also assessed the strategies participants preferred, because it is a known individual difference that influences task performance and examined whether strategy preference was modulated by emotion. Experiment 1 used a between-participant design, traditional in the WPT literature. Experiment 2 manipulated emotion within-participant, to increase power, but the results suggested that a between-participant design was favourable. Experiment 3 reverted to a between-participant design and employed a sequential Bayesian design (Schönbrodt & Wagenmakers, 2018). All experiments were preregistered on OSF (Experiment 1: https://osf.io/a7r8x/?view_only=66dd0c19718b43c9a76705321488832f, Experiment 2: https://osf.io/vyrtj?view_only=f539f2a6dd6345678da2945c9ce9ab93, Experiment 3: https://osf.io/hpkba?view_only=b39174160df34997b66a6b7aab668b88).

In addition to the key hypothesis, we also expected to replicate previous findings. Based on Schwabe and Wolf’s reasoning (2012), our second hypothesis was that in the neutral condition, the strategies participants would use most frequently would be the simple ones in the PA condition, because they rely mostly on the hippocampus, and complex ones in the FB condition, because they rely on the striatum. Finally, our third hypothesis was that for the FB version, complex strategies would be used more frequently in the negative condition compared with the neutral one.

## Experiment 1

### Method

Participants were randomly assigned to one of the four conditions (PA-negative, PA-neutral, FB-neutral, FB-negative). The method and plan of analysis of the data of Experiment 1 were preregistered (https://osf.io/a7r8x/?view_only=66dd0c19718b43c9a76705321488832f). All deviations from the preregistration are clearly stated in the method or results sections.

### Participants

A power analysis was conducted to provide an approximate sample size required (*N* = 43 per group; power = 0.9, α = 0.05) to detect a medium effect size (*f* = 0.25) in a 2x2 between-participants design ANOVA. A total of 216 participants were recruited (144 females, 3 others, 3 participants did not report their gender; age *M* = 29.29 years; *SD* = 6.08). All participants were between 18 and 40 years of age, fluent in English and were located in the UK or Ireland. Exclusion criteria included having a diagnosis of mental health disorders, medical issues such as perception or motor problems that would make participation challenging, neurological disorders, such as dyslexia or past concussions, as well as taking medication or recreational drugs that influence brain function (psychoactive drugs). A set of preregistered performance-based exclusion criteria was used (see below) to ensure high quality of data in this online study. The number of participants excluded due to each of the preregistered exclusion criteria is detailed in Table 1, leaving a final sample of 172 participants, which corresponds to the sample size required by the power analysis.

**Table 1:** Number of excluded participants in Experiment 1

**Conditions**	Missing data (more than 10%)	Instruction checks	Task checks	Self-reporting technical issues	Pretask valence ratings	Self-reporting taking notes	Accuracy	Data included out of x for each condition	% of participants included for each condition
FB-Neutral	0	2	3	2	0	1	2	43 out of 53	81%
FB-Negative	0	0	3	1	0	0	7	43 out of 54	79.6%
PA-Neutral	1	0	0	2	0	0	8	43 out of 54	79.6%
PA-Negative	0	0	1	2	0	1	8	43 out of 55	78%
**TOTAL**	**1**	**2**	**7**	**7**	**0**	**2**	**25**	**172 out of 216**	**79%**
**%**	**0.46%**	**0.9%**	**3.2%**	**3.2%**	**0%**	**0.9%**	**11.5%**		

Some participants met multiple exclusion criteria (missing data and attention checks for example) however they were assigned to one exclusion criterion following the order of the criteria in the table for clarity purposes.

Participants were recruited through the recruitment platform “Prolific.” They were reimbursed for their time (£4 for 30min). All experiments were approved by the University of Cambridge Psychology Research Ethics Committee (PRE.2022.041).

### Performance-based exclusion criteria

Participants were excluded if (1) they failed the attention instruction checks by giving more than 2 incorrect answers (out of 10), (2) they failed the attention task checks by giving more than 3 incorrect answers (out of 10), (3) they reported having encountered technical issues in a compliance survey at the end of the task, (4) their accuracy in the memory test was below 0.5, (5) their pretask valence ratings were above 5 for the negative condition and/or below 3 for the neutral condition, (6) they reported having taken notes in a compliance survey at the end of the task, (7) more than 10% of the trials were missing in their dataset (20 trials out of 200 for the learning session or 4 trials out of 42 for the test session).

### Materials

We chose to manipulate the cues and not the outcomes to avoid the interpretative difficulty that Prince and colleagues (2012) encountered, where the emotional value of an outcome that is a negatively-valence image (a picture of dangerous animals), but also represents positive feedback (“correct” response), is ambiguous.

*Cues*: Eight neutral and eight negative images were selected, which were divided into two sets of four images, an “old” set and a “new” set. Two sets of images were created to conduct habituation checks for valence and arousal at the end of the task. The old and new sets were counterbalanced across participants. The images were controlled for thematic similarity and taxonomic similarity in a previous study (Riberto et al., 2022) by only selecting images showing people outdoors either doing laundry (neutral condition) or experiencing a car accident (negative condition). The images were controlled for objective visual properties, such as luminance, contrast, and colour (Table 2). The original study reports that ten healthy participants rated the valence and the arousal of the stimuli. These ratings are also included in Table 2. Neutral images were rated as significantly different from negative images with negative images rated lower in valence, and higher in arousal, compared with neutral images. The allocation of images to cues (Table 3) was counterbalanced across participants and patterns, an increased level of control that was desired here because we used real life-event images and not tarot cards.

**Table 2:** Visual features of the stimuli used in Experiments 1 and 2

	**Emotional or neutral**				**Statistics**					
	Neutral		Emotional		Main effect of emotion		Main effect of set		Interaction emotion*set	
	Set 1	Set 2	Set 1	Set 2	F	*p*	F	*p*	F	*p*
Luminance	97.27 ± 20.49	111.22 ± 31.14	104.99 ± 10.40	116.93 ± 31.47	<1	>.10	1.07	>.10	<1	>.10
Contrast	65.41 ± 13.40	58.37 ± 10.5	66.67 ± 4.44	61.41 ± 6.78	<1	>.10	1.7	>.10	<1	>.10
R	105.45 ± 12.823	124.8 ± 28.72	113.91 ± 18.20	118.08 ± 31.4	<1	>.10	<1	>.10	<1	>.10
G	92.81 ± 23.71	109.69 ± 34.03	101.69 ± 7.51	115.15 ± 32.0	<1	>.10	1.31	>.10	<1	>.10
B	98.8 ± 25.79	83.5 ± 28.31	98.52 ± 6.18	123.12 ± 31.86	2.45	>.10	<1	>.10	2.52	>.10
Jpeg	65319 ± 17003	63184 ± 12487	67807 ± 16980	60170± 10348	<1	>.10	<1	>.10	<1	>.10
Entropy	7.63 ± .24	7.67 ± .22	7.76 ± .08	7.67 ± .15	<1	>.10	<1	>.10	<1	>.10
Valence	4.8 ± .54	5.27 ± .65	1.75 ± .40	1.7 ± .45	159.723	<.001	<1	>.10	1.00	>.10
Arousal	4.625 ± .20	4.27 ± .42	8.00 ± .27	7.9 ± .32	483.951	<.001	2.0	>.10	1.00	>.10

Images used for the cues in Experiments 1 and 2, which were controlled for luminance, contrast, Red, Green, Blue, Jpeg, Entropy. Subjective features, such as Valence and Arousal, were also compared.

**Table 3:** Learning environment in Experiment 1

*Cue*	*Images present*	*Newspaper* *Y*	*Newspaper* *Z*	*Total*	*P(cue)*	*P(Newspaper Y/cue)*	*Optimal answer*
*A*	0001	17	2	19	0.095	0.89	Y
*B*	0010	7	2	9	0.045	0.78	Y
*C*	0011	24	2	26	0.130	0.92	Y
*D*	0100	2	7	9	0.045	0.22	Z
*E*	0101	10	2	12	0.060	0.83	Y
*F*	0110	3	3	6	0.030	0.50	NA
*G*	0111	17	2	19	0.095	0.89	Y
*H*	1000	2	17	19	0.095	0.11	Z
*I*	1001	3	3	6	0.030	0.50	NA
*J*	1010	2	10	12	0.060	0.17	Z
*K*	1011	5	4	9	0.045	0.55	Y
*L*	1100	2	24	26	0.130	0.08	Z
*M*	1101	4	5	9	0.045	0.44	Z
*N*	1110	2	17	19	0.095	0.11	Z
*Total*		100	100	200	1.00		

The probability of each cue and its outcome, following previous studies (Shohamy, Myers, Onlaor et al., 2004). Each row in the column “image present” refers to the presence of cues 1 to 4; for example, in the first and second rows the cue comprises of a single image, with cue A consisting of image 4 and cue B of image 3.

*Outcomes*: For the outcome, we chose to use fictional newspaper names instead of the classic weather outcomes (rain or shine). We thought it would be more intuitive for participants to be asked to make associations between pictures of people and newspaper names as compared to rain or sun outcomes. Additionally, we wanted the outcomes to be neutral, and rain can have an emotional connotation. Therefore, two fictive newspaper names were created the “*Tuesday Courier*” or the “*Wednesday Bulletin*” and were used as the outcomes.

### Design and procedure

The experiment was conducted online using the software GORILLA (Anwyl-Irvine et al., 2020). All participants provided written informed consent.

Participants first were told that it was a learning experiment; hence, they were instructed to try to memorise the association between cues and outcomes and were told that good performance would lead to a bonus. The bonus was added after pilot studies (not reported) showed low memory accuracy. After this, participants read the general instructions and then were asked to answer two attention instruction check questions. These questions ensured that participants read the instructions carefully. A typical type of question would be to instruct participants to always answer “blue,” and then ask the question, “What’s the colour of your hair?” Ten attention instruction checks were administered throughout the task, presented after instructions or breaks.

Following two attention instruction check questions, participants completed two habituation checks. First, they performed a valence rating task. Participants saw the four images from the “old” set and the four images from the “new” set in a random fashion, one at a time and were asked to rate the emotionality of each image using a 1 to 9 Likert scale (extremely negative to extremely positive). Then, participants went through a choice-task, by showing them two images on the screen for 5 s and asking them to pick the one that appeared more neutral to them.

After this, participants were presented with the WPT (Gluck et al., 2002; Shohamy, Myers, Onlaor et al., 2004). The learning session of the task usually includes 200 trials. In each trial, participants are presented with a cue comprising of one, two, or three images. Here, the tarot card images were replaced by naturalistic images (neutral or negative). Each trial is associated with one of the two outcomes; overall, these two outcomes occur equally often. In the classic paradigm, these outcomes are “rain” and “shine” cartoons (Gluck et al., 2002; Shohamy, Myers, Onlaor et al., 2004), but here we used fictional newspaper names. As done in previous literature (Gluck et al., 2002; Shohamy, Myers, Onlaor et al., 2004), each image was independently associated with each outcome with a different but fixed probability. For instance, the probability of newspaper Y was 0.2 for image 1, 0.4 for image 2, 0.6 for image 3, and 0.8 for image 4. Therefore, image 1 was strongly predictive of the newspaper Z, image 2 was weakly predictive of the newspaper Z, image 3 was weakly predictive of the newspaper Y, and image 4 was strongly predictive of the newspaper Y. There were 14 cues in total, labelled A-N. Their frequencies as well as the probability of the outcome associated with them are shown in Table 3. The probability with which each image predicted each outcome was obtained by calculating the probability of that outcome given that the image was present P(outcome/image) divided by the total probability that the outcome would occur regardless of the presence of the image P(outcome).

At the start of the task, participants were presented with instructions inviting them to play a game in which they are a photographer submitting their photographs for publication, at either the “*Tuesday Courier*” or the “*Wednesday Bulletin*.” This cover story is conceptually identical to the traditional WPT cover story, where participants play the role of a weatherman, predicting whether it will rain or shine. For the PA version, participants were told that they would be presented with a combination of photographs as well as the name of the newspaper. They had to observe the trials and try to remember the associations between the images and the newspaper. For the FB version, participants were told that when seeing the combination of photographs, they had to guess which newspaper had accepted their photographs either the “*Tuesday Courier*” or the “*Wednesday Bulletin*,” by pressing on one of the two buttons. After answering they would receive feedback, and based on this, they would learn throughout the task the associations between the photographs and the newspapers.

After reading the instructions, participants moved on to the learning phase, including 4 blocks of 50 trials (200 trials in total) with breaks in between. On each trial, a cue was displayed made of a specific combination of images. For the FB version, two boxes displaying both newspaper names were shown. Participants were asked to click on the newspaper they believed had accepted their photographs. They had 5 s to answer (with a 3-s countdown after 2 s). Once they made their prediction, participants received immediate feedback showing the correct newspaper associated with the cue. The feedback screen was presented for 2 s. Correct responses were signalled by the text “correct answer,” as well as a smiling cartoon-like face. Incorrect responses were signalled by the text “incorrect answer,” as well as a frowning cartoon-like face. This procedure closely followed the classic procedure found in previous literature (Gluck et al., 2002; Shohamy, Myers, Onlaor et al., 2004). If participants did not respond, the incorrect answer screen was displayed, with an additional statement in red font telling participants that they missed a round and should pay attention. This part was added to monitor participants more closely as pilot studies revealed that participants sometimes missed a couple of trials.

**Fig. 1:**
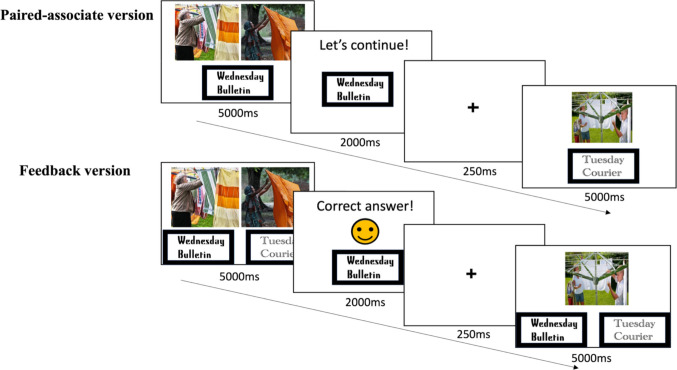
Learning session of the task for each of the two versions. Paired-associate version: Participants were presented with the cue (a combination of images) and the outcome. After this, an additional screen showing the outcome was presented. Feedback version: Participants were presented with the cue and two outcome options, and were asked to respond within a time window. After making a response, feedback was given

For the PA condition, below the cue, a box was presented displaying either “*Tuesday Courier*” or “*Wednesday Bulletin*.” Participants were asked to look at the cue and the associated newspaper and click on the newspaper’s name to move to the next trial. After clicking on the newspaper’s name, participants were presented with an additional screen for 2 s showing the newspaper’s name again and a sentence “Let’s continue.” This was to ensure similar timing with the FB version (Fig. 1). Similarly to the FB version, if the participant did not click within 2 s on the newspaper box on the first screen, a clock showing a 3-s countdown appeared. If participants did not respond within the next 3 s, the trial was terminated. Participants were then shown the additional screen showing the newspaper’s name and the sentence “Let’s continue,” but in addition to that, a statement in red font popped up saying “you missed a round, pay attention” on the same screen.

For both versions, ten attention checks were included. They consisted of trials asking participants to click on the newspaper that was shown in the previous screen (the feedback screen for the FB version and the additional screen for the PA version) out of the two newspapers.

Participants were given 20-s breaks every 50 trials, as well as a final break at the end of the learning session. Every break was followed by two attention instruction checks. After the 4 blocks of learning trials, participants were asked to complete the test phase in which their memory for the association between cues (i.e., combination of images) and outcomes was assessed. The test phase was the same in both versions of the task. Participants saw each of the 14 cues 42 trials in total, in a blocked randomised fashion. For each cue, participants were asked to indicate which newspaper was associated with it, by selecting one of the two possible newspaper names presented below the cue. No feedback was given during the test phase. The two habituation checks (valence ratings and choice-task) were repeated after the test phase.

### Statistical analysis

*Associative memory*: Following previous studies (Gluck et al., 2002; Shohamy, Myers, Onlaor et et al., 2004), in this probabilistic categorisation task, the response qualified as correct was the optimal response, namely, the outcome that was associated most often with that cue. For example, cue A occurred on 19 trials in total. In 17 trials, it was associated with the newspaper Y and in 2 trials with the newspaper Z; therefore, the optimal response was the Y. No optimal response was defined for cue F and I, because they were equally often associated with each outcome. Participants’ accuracy at the test phase was averaged across trials and then entered into a two-way ANOVA with the between-participants factors of Emotion (Neutral vs. Negative) and Task Version (PA vs. FB). Regardless of the results of the ANOVA, we preregistered to conduct four planned comparisons: between the PA and the FB versions within each emotion condition (simple effect of the Task Version factor) and between Neutral and Negative conditions within each version (simple effect of the Emotion factor).

*Strategy analyses:* Based on previous literature (Gluck et al., 2002; Lagnado et al., 2006; Newell et al., 2007), we investigated response strategies, by generating model response profiles based on how an ideal participant would respond on each trial if they had been following one of the two strategies:

1. Complex: Multicue maximizing strategy or Multicue matching strategy. Participants attend to all images on each trial and respond either with the optimal response (the outcome most often associated with that cue) or according to the distribution of outcomes associated with each image in the cue, respectively (e.g., for cue M in Table 3, maximising means always selecting newspaper Z, and matching means selecting Y 44% of the time).
2. Simple: Singleton or one-image strategy. In the former, participants learn the outcomes for trials with a single image and respond randomly to trials with multiple images. In the latter, participants respond to each trial based on the presence or absence of a single image, regardless of the other images.

For each participant, the degree to which each ideal mathematical model fit the participant’s data was computed, minimising the mean square error. The lowest score was used to indicate the best model that fit each participants’ data. The model that most closely fit a participant’s individual response profile indicated the strategy that the participant most likely used. The frequency of complex and simple strategies was then computed across participants for each condition.

To quantify the fit, as explained in Gluck et al. (2002), the squared difference between the number of “newspaper Y” responses generated by a participant and the number predicted by a model, summed across all cues was computed. This score was normalised by dividing the sum of squares of the total presentations of each cue:

$$\text{Score for Model M }= \frac{{\sum }_{p}({\#newspaperY\_expected}_{P,M}-{\#newspaperY\_actual}_{P}{)}^{2} }{{\sum }_{p}({\#presentations}_{P}{)}^{2}}$$

in which *P* = cue A...N; *#presentations*_*P*_ is the number of times cue *P* appears in the test session (3 times); *#newspaperY_expected*_*P,M*_ is the number of newspaper Y responses expected to cue *P* under model *M*, and *#newspaperY_actualp* is the actual number of newspaper Y responses the participant made to cue *P* during the test session.

Chi-square analyses were conducted to determine whether the distribution of best-fit strategies differed between negative and neutral conditions as well as between learning versions.

*Manipulation checks - Valence ratings:* First, we checked whether valence ratings at the start of the experiment were significantly different between the two emotional conditions by running a 2-way ANOVA with between-participants factors of Emotion (Neutral vs. Negative) and Task Version (PA vs. FB) with pretask valence ratings as the dependent variable. Additionally, to check whether participants habituated during the course of the experiment and how this may have affected their emotional response to the cards, two 3-way ANOVA analyses were conducted with the between-participants factors of Emotion (Neutral vs. Negative), Task Version (PA vs. FB), and the within-participants factor of Image-type (Old vs. New). The first examined effects on posttask valence ratings, as if habituation occurred, the posttask ratings of old images should be less negative than those of new images. The second examined effects on the difference between pretask and posttask valence ratings. If habituation occurred, the difference should be bigger for old compared with new images.

*Manipulation checks - Choice-task:* Habituation would be evident in increased proportion of old images picked in the choice-task posttask as compared to pretask. This difference was entered as the dependent measure in a 2-way ANOVA with between-participant factors of Emotion (Neutral vs. Negative) and Task Version (PA vs. FB).

### Results

#### Manipulation checks

**Fig. 2:**
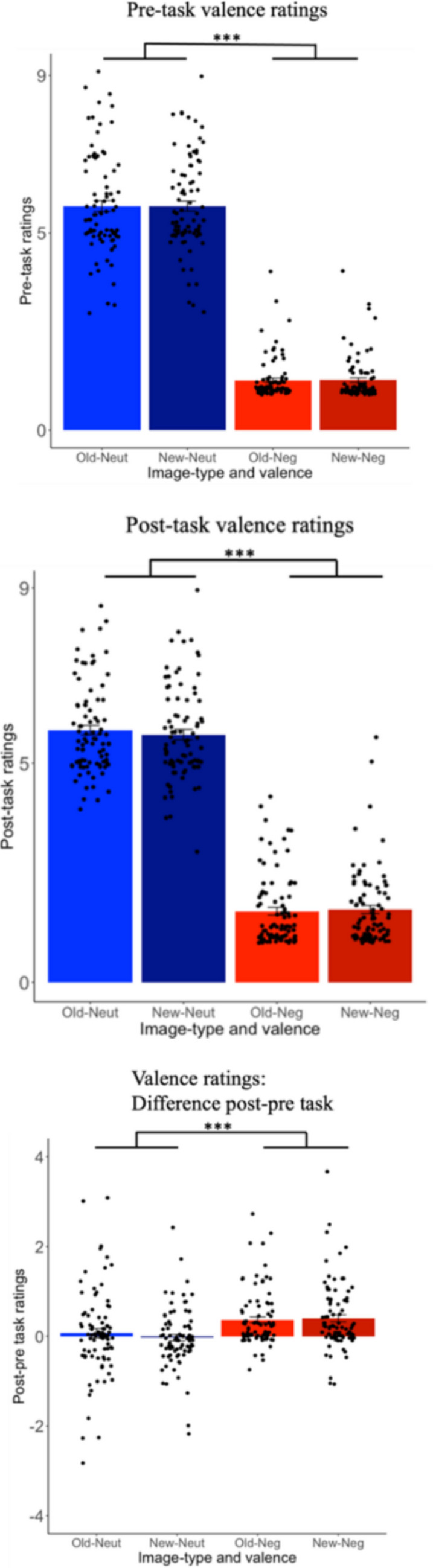
Results of the manipulation checks in Experiment 1. Using a 1 to 9 Valence scale, the plots depict Pretask (top row) and Posttask (middle row) valence ratings, as well as the difference between them (bottom row), for images viewed during the WPT (old) and those that were rated once prior to the WPT but not viewed afterwards (new). ***p < 0.001.

Valence ratings: Valence ratings supported the allocation of images to the negative and neutral conditions, because for both pretask and posttask, the only significant effect was more negative valence ratings of images allocated to the negative condition. The analysis of pretask ratings showed a main effect of Emotion, *F*(1, 168) = 988.963, *p* < 0.001, *η*_*P*_^*2*^ = 0.855, due to lower pretask valence ratings for the negative condition (mean [*M*] = 1.26, standard deviation [*SD*] = 0.49) as compared to the neutral condition (*M* = 5.68, *SD* = 1.20). No effect of Task Version and no interaction of Task Version*Emotion was significant (*p* values > 0.289). The analysis of posttask ratings showed a main effect of Emotion, *F*(1, 336) = 1572.314, *p* < 0.001, *η*_*P*_^*2*^ = 0.824, due to lower ratings for the negative condition (*M* = 1.64, *SD* = 0.84) compared with the neutral condition (*M* = 5.70, *SD* = 1.03). No effects of Task Version and Image-set were found (*p* values > 0.620). No interactions between Image-set*Task Version, Image-set*Emotion, Task Version*Image-set, Task Version*Image-set*Emotion were found (*p* values > 0.207; Fig. 2).

The analysis of the post-pre task valence ratings difference revealed a main effect of Emotion, *F*(1, 336) = 19.58, *p* < 0.001, *η*_*P*_^*2*^ = 0.055, showing increased difference of ratings for negative condition (*M* = 0.38, *SD* = 0.68) compared with neutral condition (*M* = 0.017, *SD* = 0.83). No effects of Task Version and Image-set were found (*p* values > 0.112). No interactions between Image-set*Task Version, Image-set*Emotion, Task Version *Image-set, Task Version*Image-set*Emotion were found (*p* values > 0.416). The main effect of Emotion indicated that the difference post-pre task ratings was larger for negative compared to neutral images, suggesting that negative images were rated as slightly more positive at the end relative to the start of the task, compared to neutral images, as can be seen in Figure 2.

*Choice-task*: The difference between the proportion of old items selected at the end of the task versus at the start in the choice-task was investigated. The test showed no effect of Task Version, no effect of Emotion and no interaction between Task Version*Emotion (all *p* values > 0.207).

#### Associative memory accuracy

**Fig. 3:**
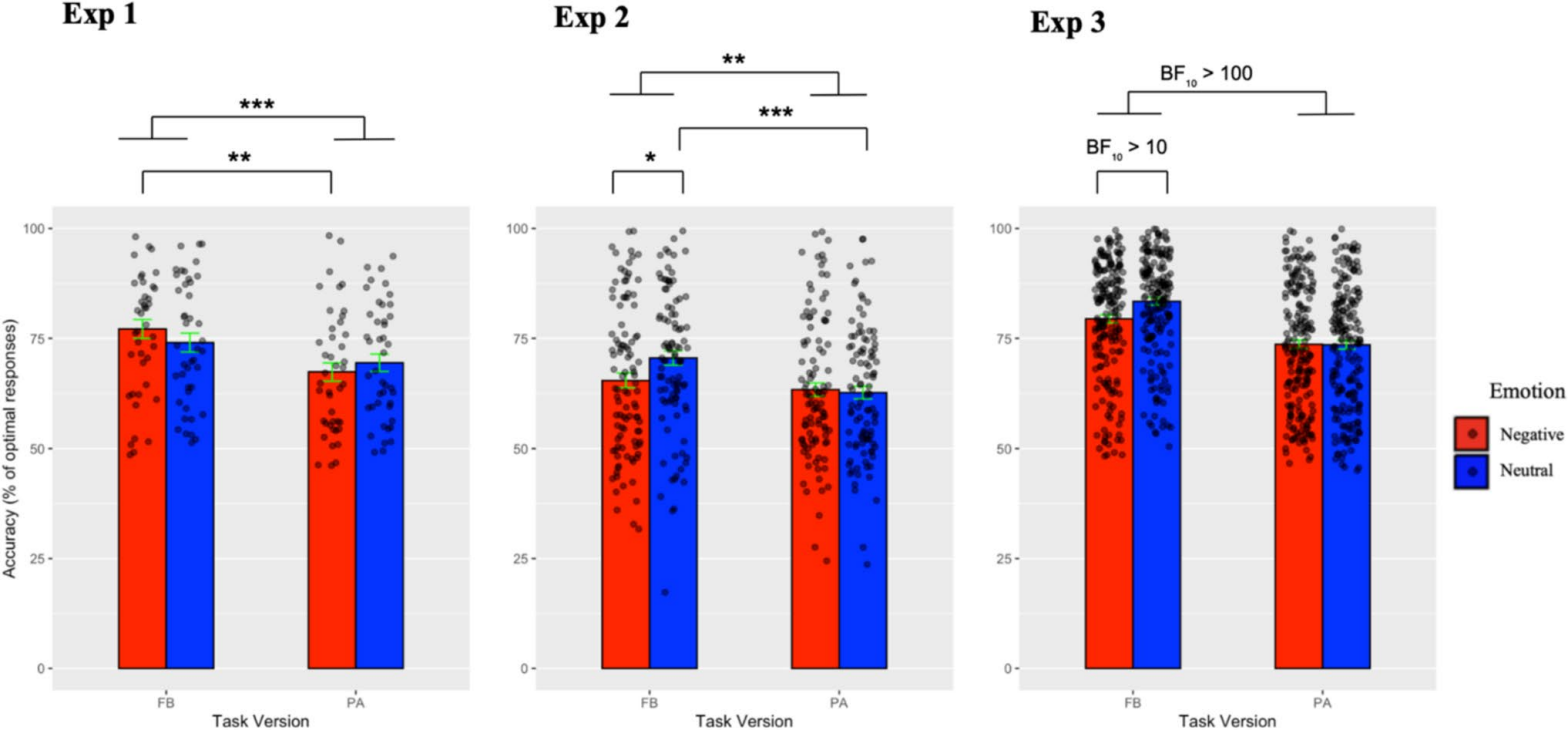
Associative memory performance across the four conditions of the three experiments. Bar plots display group means of proportion of optimal responses, with standard error markers and overlaid individual participant datapoints. *p < 0.05; **p < 0.01; ***p < 0.001; BF_10_ indicates evidence in favour of the alternative hypothesis.

Figure 3 (left) shows accuracy for the four groups. A 2-way ANOVA with between-participants factors of Emotion (Neutral vs. Negative) and Task Version (PA vs. FB) showed a main effect of Task Version, *F*(1,168) = 11.864, *p* < 0.001, *η*_*p*_^*2*^ = 0.07, with higher memory performance for the FB version compared to the PA version. No effect of Emotion was found, *F*(1,168) = 0.062, *p* = 0.804, *η*_*p*_^*2*^ = 0.00, nor Task Version*Emotion interaction, *F*(1,168) = 1.541, *p* = 0.216, *η*_*p*_^*2*^ = 0.009. A numerical effect of Task Version was present in both neutral and negative conditions, which planned comparisons showed was significant in the negative condition, *t*(84) = 3.290, *p* = 0.001, *d* = 0.709, but did not reach significance for the neutral condition, *t*(84) = 1.569, *p* = 0.12, *d* = 0.338 (Fig. 3, left). The planned comparison between negative and neutral conditions for the PA version showed no effect of Emotion, *t*(84) = 0.728, *p* = 0.469, *d* = 0.157. Similar results were found for the FB version, *t*(84) = 1.02, *p* = 0.311, *d *= 0.22.

#### Strategy use

**Fig. 4:**
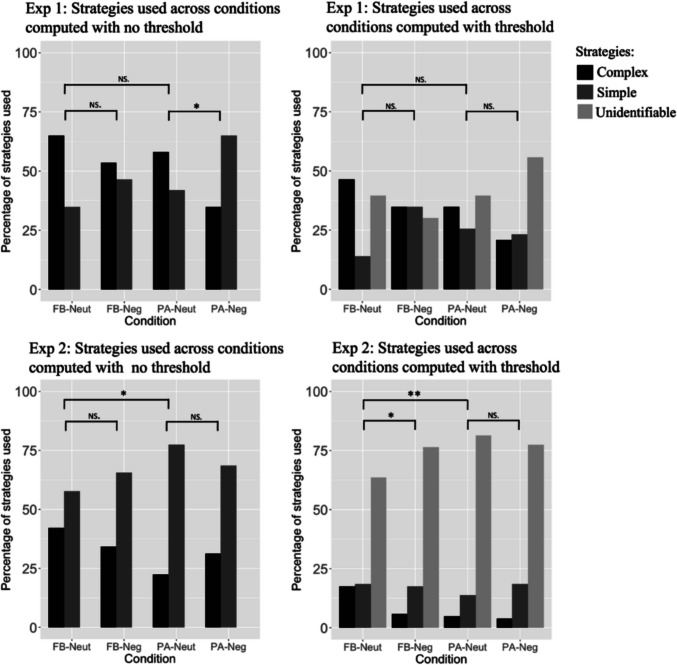
Strategies used across conditions in Experiment 1 (top row) and Experiment 2 (bottom row). The left column depicts the percentage of strategies used across conditions when computed with no threshold, according to the preregistration. The right column depicts the same when strategy use was computed with threshold, a nonregistered analysis. NS = nonsignificant; *p < 0.05; **p < 0.01.

Figure 4 (top left) shows the percentage of participants in each group using each strategy. The results suggested increased use of simple strategies in the PA negative condition, compared with all other conditions. Collapsing across emotional conditions, there was a numerical trend towards the expected greater proportion of participants using complex strategies in the FB condition (59.3%) than in the PA condition (46.5%), though the difference did not reach significance, χ^2^(1) = 2.823, *p *= 0.093. Contrary to expectations however, when collapsing across Task Version, there was evidence that participants used simple strategies more often in the Negative condition (55.8%) than Neutral condition (38.4%), χ^2^(1) = 5.250, *p* = 0.022. This bias towards simple strategies for Negative emotion was significant in the PA version alone, χ^2^(1) = 4.674, *p* = 0.031, but not the FB version alone, χ^2^(1) = 1.204, *p* = 0.272. When looking at Neutral or Negative conditions alone, any difference between Simple and Complex strategies did not reach significance for Neutral conditions, χ^2^(1) = 0.443, *p* = 0.506, nor Negative conditions, χ^2^(1) = 3.018, *p* = 0.082.

Because our results regarding strategies differ from Prince and colleagues (2012), Schwabe and Wolf (2012), and Thomas and LaBar (2008), who found that more complex strategies were used in the Negative condition, we decided to run additional analyses that resembled theirs more closely. Following two of these authors and other previous papers (Gluck et al., 2002), if the model fit was not sufficient, a third category of “unidentifiable” strategy was assigned (Fig. 4, top right). An insufficient fit was based on a model fit score of more than 0.1. Similar chi-square analyses were run as the ones presented above, but now across all three strategy types. Although these tests did not reveal any significant strategy differences for any of our planned comparisons, χ^2^(2)’s < 5.770, *p* values > 0.056, there was still a numerical trend for Complex strategies being used less often in the Negative than Neutral groups, for both Task versions, opposite to our hypothesis from the literature.

#### Strategy use and associative memory performance

**Fig. 5:**
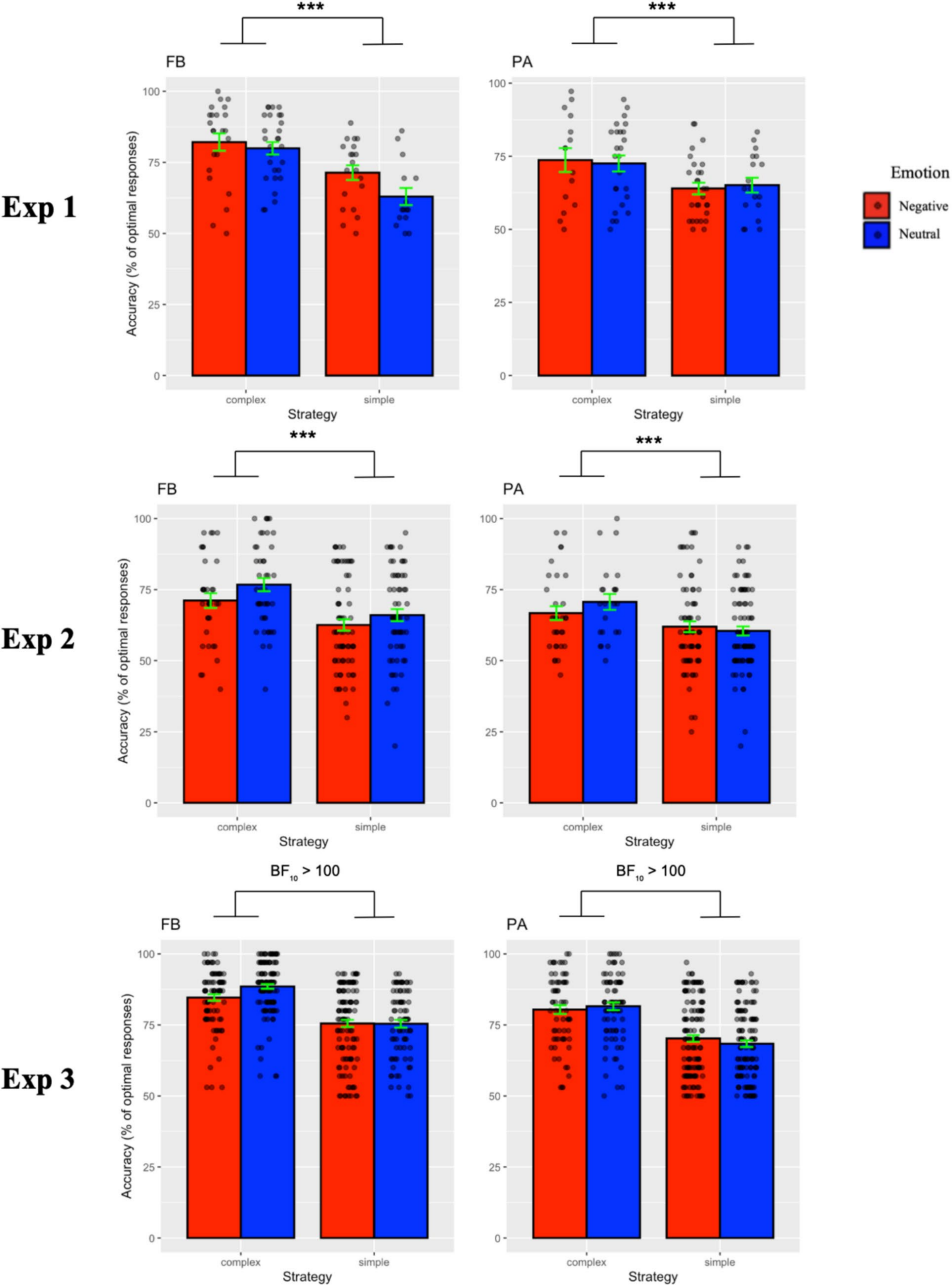
Associative memory accuracy by Task Version, Strategy Use, and Emotion Condition across all three experiments. ***p < 0.001, BF_10_ > 100 indicates decisive evidence in favour of the alternative hypothesis.

Participants were more accurate in the PA version when they used complex strategies (Fig. 5, top). A 3-way ANOVA was run with the between-participants factors of Task Version (FB vs. PA), Emotion (Neutral vs. Negative), and Strategy (Complex vs. Simple). Similar to the previous analyses, a main effect of Task Version was found, *F*(1, 164) = 7.177, *p* = 0.008, *η*_*P*_^*2*^ = 0.042, due to increased memory accuracy for FB compared with PA. Additionally, a main effect of Strategy was found, *F*(1,164) = 32.574, *p* < 0.001, *η*_*P*_^*2*^ = 0.166, revealing increased accuracy when participants used complex strategies (*M* = 0.774, *SD* = 0.14) compared with the use of simple strategies (*M* = 0.658, *SD* = 0.113). No main effect of Emotion was found, *F*(1,164) = 1.818, *p* = 0.179, *η*_*P*_^*2*^ = 0.011. No interactions were significant (*p* values > 0.180).

## Discussion

The pattern of WPT performance accuracy in the four conditions did not support our key hypothesis. According to DRT, negative emotional arousal should impair hippocampal associative learning, which should in turn affect the PA version of the WPT to a greater extent than the FB version. However, we did not obtain evidence for a greater detrimental effect of negative stimuli on accuracy in the PA compared to the FB condition, and accuracy was not decreased in the negative compared with the neutral PA conditions. Although the accuracy in the negative PA condition was lower than in the negative FB condition, this effect could have been due to the overall decreased performance in the PA condition. We delay interpreting these findings to the General Discussion.

The results did not support our secondary hypotheses regarding strategies either. Based on previous claims that the hippocampal system favours simple strategies (of relying on just one cue), we expected to see less use of such strategies for negative cues, particularly in the PA version. Although we did find a numerical trend for simple strategies to be used more often than complex ones in the neutral PA condition compared with the neutral FB condition, as might be expected from previous WPT literature, we did not find evidence that simple strategies were more common for negative than neutral stimuli in the FB version. Indeed, if anything, we found the opposite pattern of negative stimuli inducing the use of complex strategies more than neutral ones.

Note that the validation checks confirmed that participants found the negative stimuli more negative than neutral ones, even at the end of the experiment (with negligible habituation). In Experiment 2 we attempted to increase power to detect detrimental effects of emotion, by manipulating this factor within-participants.

## Experiment 2

### Method

Experiment 2 sought to increase statistical power for the comparison of Negative versus Neutral stimuli by manipulating this within-participant. This resulted in a mixed design with the Task Version (PA vs. FB) remaining between-participants. Experiment 2 used the same cues and outcomes as Experiment 1, although two additional outcomes had to be created because participants saw both negative and neutral cues. The methodology was identical other than minor changes, introduced to reduce the length of the experiment. The method and plan of analysis of the data of Experiment 2 were preregistered (https://osf.io/vyrtj/?view_only=f539f2a6dd6345678da2945c9ce9ab93). Any deviations from the preregistration are stated in the *Methods* or *Results* sections.

#### Participants

In contrast to Experiment 1, we decided to use Brysbaert’s suggestions (2019) instead of using the classic power analysis computation. We wanted to be able to detect smaller effect sizes, because Experiment 1 revealed some significant differences in planned contrasts but not in ANOVA interactions. As suggested by Brysbaert (2019), two groups of participants of *N* = 100 would allow us to detect a significant interaction at *p* < .05, in addition to a significant one-tailed Bonferroni-corrected main effect in one group and not the other, for a two-way ANOVA with one repeated-measures factor and one between-groups factor, with a *d* = .4 in one group (not the other) and 80% power. A total of 265 participants were recruited (174 females; age *M* = 30.04 years; *SD* = 6.00; one participant’s age was not computed due to a typographical error in their response). Similar exclusion criteria as Experiment 1 were used. A set of preregistered performance-based exclusion criteria was used (see below) to ensure high quality of data in this online study. The number of participants excluded due to each of the preregistered exclusion criteria is detailed in Table 4, leaving a final sample of 204 participants. Similar recruitment, reimbursement and ethical approval was employed as in Experiment 1.

**Table 4:** Number of participants excluded in Experiment 2

Conditions	Missing data (more than 10%)	Instruction checks	Task checks	Self-reporting technical issues	Self-reporting taking notes	Accuracy	Data included out of x for each condition	% of participants included for each condition
FB	0	0	7	6	1	16	102 out of 132	77%
PA	1	1	0	3	1	25	102 out of 133	76.6%
**TOTAL**	**1**	**1**	**7**	**7**	**2**	**41**	**204** **out 265**	**76.8%**
**%**	**0.37%**	**0.37%**	**2.6%**	**2.6%**	**0.75%**	**15.5%**		

Some participants met multiple exclusion criteria (missing data and attention checks for example); however, they were assigned to one exclusion criterion following the order of the criteria in the table for clarity purposes.

#### Performance-based exclusion criteria

Exclusion criteria were similar to Experiment 1, except that Experiment 2 did not include valence ratings, so exclusion criterion number 5 could not be used. Additionally, Experiment 2 used a different number of trials to Experiment 1; hence the exclusion criterion of the 10% of trials missing was adapted: when 20 of 202 trials for the learning session or 6 of 56 trials for the test session were missing, participants were excluded.

#### Materials

*Cues*: The two sets composed of four images (called “old set” and “new set”) created for Experiment 1 were used for PA and FB conditions. The two sets were counterbalanced across conditions.

*Outcomes*: In addition to the two fictive newspaper names created for Experiment 1, two additional outcomes were created for Experiment 2 (“Friday Roundup,” “Thursday Express”) as participants saw four different outcomes in total (two for the negative condition and two for the neutral condition). The four outcomes were counterbalanced across conditions.

#### Design and procedure

These were similar to Experiment 1, with the following exceptions. In Experiment 2, the valence ratings and choice-task were not included. Participants saw a total of 204 trials in the learning phase, 102 neutral and 102 negative trials, which were interleaved. In addition, four outcomes were possible in total, two per emotional condition. As in Experiment 1, each trial showed a cue made up of one, two, or three images and was associated with one of the two outcomes. Because the number of trials in Experiment 2 was reduced per emotional condition to avoid having an incredibly long task, the probability table was not the same as the one used for Experiment 1; however, we still used a probability table from previous literature (Newell et al., 2007). The frequencies of the cues as well as the probability of the outcome associated with them are shown in Table 5. Similar to Experiment 1, the images of the cues were counterbalanced across participants.

**Table 5:** Learning environment in Experiments 2 and 3

*Cue*	*Images present*	*Newspaper Y*	*Newspaper Z*	*Total*	*P(cue)*	*P(Newspaper Y/cue)*	*Optimal answer*
*A*	0001	9	1	10	0.098	0.900	Y
*B*	0010	4	1	5	0.049	0.800	Y
*C*	0011	12	1	13	0.127	0.923	Y
*D*	0100	1	4	5	0.049	0.200	Z
*E*	0101	5	1	6	0.058	0.833	Y
*F*	0110	2	2	4	0.040	0.500	NA
*G*	0111	8	1	9	0.088	0.899	Y
*H*	1000	1	9	10	0.098	0.100	Z
*I*	1001	2	2	4	0.040	0.500	NA
*J*	1010	1	5	6	0.058	0.167	Z
*K*	1011	2	2	4	0.040	0.500	NA
*L*	1100	1	12	13	0.127	0.077	Z
*M*	1101	2	2	4	0.040	0.500	NA
*N*	1110	1	8	9	0.088	0.111	Z
*Total*		51	51	102	1.00		

The probability of each cue and its outcome, used in previous studies (Newell et al., 2007).

In addition, the instructions were slightly modified to mention the four newspaper outcomes “*Tuesday Courier*”; “*Wednesday Bulletin*”; “*Thursday Express*”; or “*Friday Roundup*.” Before starting the experiment, participants did four practice trials, two with negative cues and two with neutral cues. The images used for the practice trials were of a different nature from the ones used in the task (negative images showed poverty scenes and neutral images showed people on their phones). The outcomes used in the practice trials were not used in the main task but were also newspaper names. For the main task, participants saw 102 trials for each emotional condition (hence 204 in total, 51 per block) instead of 200 in Experiment 1 and they were given a 20-s break every 51 trials (in Experiment 1, they were given a break every 50 trials). Similar to Experiment 1, participants were given a final break at the end of the learning session, and every break was followed by two attention instruction checks. With regards to the memory test phase, it was similar to Experiment 1 except that in Experiment 2, each cue was presented twice (in Experiment 1 each cue was presented three times), again for study length concerns. Therefore, for the memory test, participants saw each of the 14 cues (i.e., combination of images) of each emotional condition twice, hence 56 trials in total, in a blocked randomised fashion.

#### Statistical analysis

These were similar to Experiment 1 with the following exceptions. The optimal response computation as the probability of each outcome given a cue was different: no optimal response could be defined for cues K, M, in addition to F and I (which was already the case in Experiment 1) because these cues were equally paired with both outcomes. Other differences from Experiment 1 in the analyses were due to the mixed design. For that reason, to analyse associative memory, the planned comparisons included independent t-tests when comparing the two learning conditions, but when comparing the two emotional conditions, paired *t*-tests were used. Similarly, for the strategy analyses, as for Experiment 1, chi-square analyses were conducted. However, there was a deviation from the preregistration for the comparison of the proportion of strategies between the two emotional conditions: a McNemar test was conducted instead of a chi-square test as the data were paired, and no continuity correction was used.

### Results

#### Associative memory performance

A 2-way ANOVA with within-participants factor of Emotion (Neutral vs. Negative) and between-participants Task Version (PA vs. FB) showed a main effect of Task Version, *F*(1,202) = 8.6, *p* = 0.004, *η*_*p*_^*2*^ = 0.04*.* Similar to Experiment 1, no effect of Emotion was found, *F*(1,202) = 2.297, *p* = 0.131, *η*_*p*_^*2*^ = 0.011; however, in contrast to Experiment 1, a Task Version*Emotion interaction was found, *F*(1,202) = 3.969, *p* = 0.048, *η*_*p*_^*2*^ = 0.019 (Fig. 3, middle). The planned comparison for the neutral condition showed a significant increase in accuracy for FB learning compared to PA learning, *t*(202) = 3.557, *p* < 0.001, *d =* 0.498, an effect that was apparent as a numerical, nonsignificant trend in Experiment 1. In contrast to Experiment 1, the same analysis for the negative condition did not show a significant difference between the two task versions, *t*(202) = 0.922, *p* = 0.358, *d* = 0.129. In contrast to Experiment 1, the planned comparison between negative and neutral conditions for the FB version showed higher accuracy for the neutral condition, *t*(101) = 2.47, *p* = 0.015, *d =* 0.245, compared to the negative condition. For the PA version, similar to Experiment 1, no difference was found between the two Emotion conditions, *t*(101) = 0.33, *p* = 0.361, *d *= −0.34.

#### Strategy use

In this experiment, we observed increased use of simple strategies in the neutral PA version, which aligns with our hypothesis. Collapsing across Task Version, the McNemar test showed no relationship between Strategy and Emotion, χ^2^(1) = 0.011, *p* = 0.914. Among the FB conditions, the McNemar test again showed no relationship between Emotion and Strategy, χ^2^(1) = 1.6, *p* = 0.206. Similar results were found among participants of the PA condition, χ^2^(1) = 1.723, *p* = 0.189. These results contrast with Experiment 1, where participants in the negative condition version were more prone to use simple strategies compared to the neutral condition, and where more participants in the negative PA condition used simple strategies compared to the neutral condition. Lastly, in contrast to Experiment 1, chi-square test showed that among participants in the neutral condition, a higher proportion used simple strategies in the PA version (77.5%) compared with participants in the FB version (57.8%), χ^2^(1) = 8.959, *p* = 0.003. No such relationship was found among participants in the negative condition, χ^2^(1) = 0.2, *p* = 0.655.

As in Experiment 1, we conducted a more conservative, but nonregistered analysis where we computed the strategies using a threshold of 0.1; hence, participants could be using complex, simple or unidentifiable strategies. We found an increased percentage of participants who used unidentifiable strategies in this experiment compared with Experiment 1, with only 20 participants remaining who never used unidentifiable strategies. Similar tests were run as above, but a McNemar-Bowker test was used instead of McNemar to allow Strategy to have three levels. Among the PA condition, no relationship between Emotion and Strategy was found, χ^2^(3) = 0.867, *p* = 0.833, but among FB, significant relationship was found, χ^2^(3) = 8, *p* = 0.046, reflecting decreased use of complex strategies and increased use of unidentifiable strategies in the negative condition. Among participants in the neutral condition, chi-square test revealed an effect of Task version, χ^2^(2) = 10.295, *p* = 0.006, due to decreased use of complex strategies and increased use of unidentifiable strategies in PA. No such relationship was found among participants in the negative condition, χ^2^(2) = 0.433, *p* = 0.805.

#### Strategy use and associative memory performance

The preregistration included an exploratory analysis that included the factor Strategy, but did not specify what form that analysis would take. Because the same participant could use a different strategy in the negative and the neutral condition, we could not use Strategy as a between-subject factor, as in Experiment 1. We also could not use it as a within-participant factor because it was not fully crossed with the factor Emotion. To better understand the interaction between emotion, accuracy, and strategy, we conducted two 2-way ANOVAs, with the between-participants factors of Strategy and Task Version, one in the neutral and one in the negative condition.

In the neutral condition we found a main effect of Strategy, *F*(1, 200) = 20.33, *p *< 0.001, *η*_*p*_^*2*^ = 0.092, showing increased accuracy when participants used complex strategies as opposed to participants using simple strategies (Fig. 5, middle). A main effect of Task Version was also found, *F*(1, 200) = 6.312, *p* = 0.013, *η*_*p*_^*2*^ = 0.031, showing increased accuracy in the FB version compared to the PA version. No interaction between Neutral strategy* Task Version was found, *F*(1, 200) = 0.012, *p* = 0.091, *η*_*p*_^*2*^ = 0.00.

In the negative condition we also found a main effect of Strategy, *F*(1, 200) = 8.198, *p* = 0.005, *η*_*p*_^*2*^ = 0.039, showing increased accuracy when participants used complex strategies compared with those using simple strategies. No main effect of Task Version, nor interaction between Negative strategy and Task Version, was found to be significant (all *p*’s>0.283). To sum up the last two analyses, participants’ accuracy was increased if they used complex strategies, an effect which was modulated by Task Version only in the neutral condition.

### Discussion

Despite embedding Emotion as a within-participant factor, we confirmed our second hypothesis; participants in the neutral condition used more complex strategies in the FB than the PA condition, replicating the mainstream WPT literature. Crucially, the predictions derived from the DRT were not supported. As in Experiment 1, the results of Experiment 2 did not support the key hypothesis that negative emotional arousal will selectively decrease hippocampal-dependent memory, because there was no evidence for decreased memory accuracy in the negative compared to the neutral PA version. Additionally, participants used simpler strategies more often in the negative than the neutral FB condition, contradicting the third hypothesis. We discuss the implications of these key results in the General Discussion.

Only a few studies have employed a within-participants/mixed design using the WPT (Gabay et al., 2015; Holl et al., 2012; Wilkinson et al., 2008), with Task Version (PA vs. FB) manipulated within-participants. To the best of our knowledge, no previous study has used the factor Emotion as a within-participants factor using the WPT, with some arguing that, due to the probabilistic nature of the task and strong practice effects, a within-participants (or mixed) design is not feasible (Prince et al., 2012). While some results were consistent across Experiments 1 and 2, such as increased accuracy for the FB version compared with PA, as well as increased accuracy when using complex strategies, the large percentage of participants who used unidentifiable strategies suggests that the WPT is best used with a between-participants design. Indeed, only 20 participants out of 204 remained when we eliminated participants who used an unidentifiable strategy for either the negative or the neutral conditions. This proportion of unidentifiable strategies is far higher than in Experiment 1, as well as with in previous literature (Gluck et al., 2002). We conclude that the information load of the within-participant design was too high, because participants were asked to process eight cues and four outcomes, instead of four cues and two outcomes, as in the classic version.

Given the challenges of the within-participant design, it is possible that our failure to support the DRT represents a false negative. Experiment 3 was conducted to address this possibility. It was designed to test the same hypotheses but used a more rigorous statistical design that maximised chances of obtaining conclusive evidence to support or refute them.

## Experiment 3

### Method

The experimental design and methodology followed that of Experiment 1, unless otherwise noted. Like Experiment 1, this experiment used a fully between-participant design. The most important changes were as follows. First, that we used a sequential design to decide on when to stop data collection. Second, given that data from the previous two experiments did not support our hypotheses, we used Bayesian statistics to be able to provide evidence for or against the null hypothesis. In the previous two experiments, all negative cues were pictures from one semantic category (accidents), and all neutral cues were pictures from one other semantic category (laundry). For generalisation purposes, Experiment 1 included two sets of cues for each emotion condition, but both were drawn from the same semantic category. The third change we introduced in Experiment 3, again for generalisation purposes, was that we used two different semantic categories for the negative cues (accidents and poverty), and two different semantic categories for the neutral cues (laundry and phone). As in Experiment 1, each participant saw cues drawn from a single semantic category, and cue category was counterbalanced across participants. The methodology was preregistered (https://osf.io/hpkba?view_only=b39174160df34997b66a6b7aab668b88). All deviations from the preregistration are clearly stated in the method or results sections.

#### Sequential design & stopping rules

We followed a Bayesian sequential “max N” design, where data were collected until we found a BF_10_ > 6 in favour of the 2x2 interaction, a BF_10_ < 1/6 that would favour the null hypothesis of no interaction, or when a maximum N of 200 per group (800 in total) was reached. To determine the power of this design, we ran simulations using the *anovaBF* function in the BayesFactor R package (see statistics section below), with an initial group size of 24 participants, followed by batches of 8 per group up to the maximum* N* = 200 per group. Even assuming a small effect size for the interaction of Cohen’s f^2^ = 0.02, simulations showed a 92% probability of finding BF_10_ > 6 (and only 2.5% of false negatives when BF_10_ < 1/6). If the null hypothesis of no effect is true instead (f^2^=0), the probability of finding BF_10_ < 1/6 was 81% (with a false positive rate of less than 0.1%). The R code for these simulations is available, along with relevant data and analysis code below, on OSF storage (https://osf.io/das37/files/osfstorage?view_only=b39174160df34997b66a6b7aab668b88).

#### Participants

A total of 1096 participants were recruited (542 female, 8 other, 11 did not report gender, age *M* = 28.12, *SD* = 5.57). Exclusion and inclusion criteria, as well as participant recruitment and reimbursement followed Experiment 2 (Table 6). Excluded participants were replaced to reach a target of *N* = 200 per group in a 2-by-2 factorial design. The final sample of a total of *N* = 800 participants included 360 females, 5 other, 5 did not report gender, and had a mean age of 27.95 (*SD* = 5.52). The average and standard deviation of their age was similar across conditions, and approximately the same number of participants identified as female across conditions (FB-neg *M* = 27.76, *SD* = 5.38, 85 females; FB-neut *M* = 28.47, *SD* = 5.43, 105 females; PA-neg *M* = 27.57, *SD* = 5.63, 104 females; PA-neut *M* = 27.98, *SD* = 5.65, 95 females).

**Table 6:** Participant exclusion criteria and number of participants excluded in Experiment 3

Exclusion	Task attention	Instructional attention	Technical issues / notes	Performance < 0.5	Strategy unidentifiable	Missing data	Total
**N**	100	8	3	140	45	0	296

Participants were excluded if 1) they failed the instructional attention check by giving more than 2 incorrect answers (out of 6); 2) they failed the task attention check by giving more than 3 incorrect answers (out of 6); 3) they reported having encountered technical issues or having taken notes in a survey; 4) their performance in the memory test was below 0.5; 6) their strategy was categorised as “unidentifiable” based on a too low model fit (see section “Strategy use” in exp. 3). All excluded participants were replaced, to reach a total of 200 per condition.

**Fig. 6:**
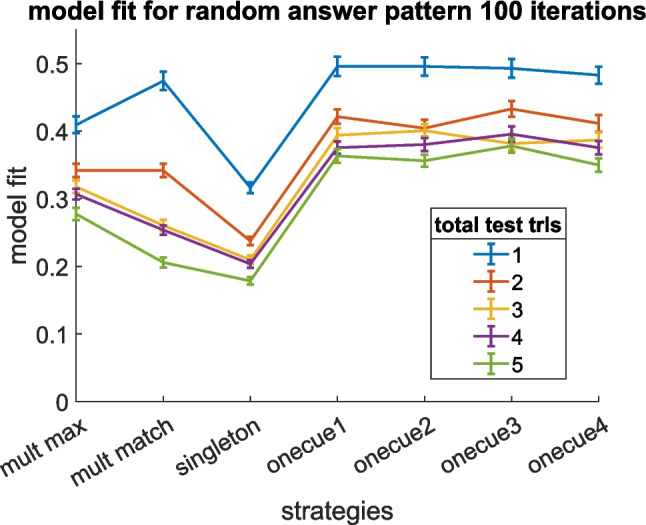
Simulations modelling strategy fit for a random response pattern. A random response pattern (selecting outcome newspaper Y or Z randomly) was generated across test trials, then the fit for each strategy was calculated following the procedure described in strategy analyses (see Exp. 1). This procedure was repeated N=2000 times, to calculate the mean model fit for each strategy based on a random response pattern. Model fit is shown as a function of strategy when varying the amount of test trials per pattern (colour coded lines). Mean and standard deviation are shown

The number of trials in the learning phase followed Experiment 2 with 102 trials (instead of 200 in Experiment 1). We halved the number of learning trials compared to Experiment 1, both because performance in Experiment 2 was good enough, and because the earlier stages of the WPT are more sensitive to the difference between hippocampal and striatal processes (Hopkins et al., 2004). The test phase, in which participants classified cues (i.e., combination of images) with their associated outcomes, followed Experiment 1 with 42 trials (3 per cue), based on new simulations of what chance performance looks like (see ‘Strategy analysis’ and Fig. 6).

#### Materials

For generalisation purposes, here we used four stimulus sets - two emotional sets and two neutral sets. For the neutral condition, the cues depicted either people hanging up laundry or talking on the phone (laundry or phone set, respectively). For the negative condition, cues depicted scenes of car accidents or poverty. Each participant encountered a single stimulus set, and stimulus sets were counterbalanced between participants. There were no significant differences between the visual properties of the two stimulus sets, but the negative and neutral sets differed on valence and arousal, as expected (Table 7).

**Table 7:** Visual features of the stimuli used in Experiment 3

	Emotional or neutral				Statistics					
	Neutral		Emotional		Main effect of emotion		Main effect of set		Interaction emotion*set	
	Set 1	Set 2	Set 1	Set 2	F	*p*	F	*p*	F	*p*
**Luminance**	107.67 ± 35.43	97.38 ± 20.38	96.78 ± 14.77	102.81 ± 15.90	<1	>.10	<1	>.10	<1	>.10
**Contrast**	61.99 ± 16.21	66.03 ± 12.26	50.97 ± 10.53	63.54 ± 4.13	1.34	>.10	2.04	>.10	<1	>.10
**R**	106.31 ± 36.83	105.73 ± 12.41	108.56 ± 9.33	102.40 ± 15.40	<1	>.10	<1	>.10	<1	>.10
**G**	108.60 ± 34.23	93.58 ± 22.92	93.20 ± 17.58	102.84 ± 16.08	<1	>.10	<1	>.10	1.07	>.10
**B**	106.36 ± 38.02	95.01 ± 30.85	84.32 ± 16.46	103.67 ± 17.85	<1	>.10	<1	>.10	1.26	>.10
**Jpeg**	99332.50 ± 41140	63568.25 ± 18203	64232.00 ± 11620	61134.50 ± 4779	2.58	>.10	2.77	>.10	1.96	>.10
**Entropy**	7.54 ± 0.30	7.66 ± 0.21	7.42 ± 0.27	7.62 ± 0.17	<1	>.10	1.69	>.10	<1	>.10
**Valence**	5.03 ± 0.17	4.95 ± 0.66	2.60 ± 0.34	2.10 ± 0.57	123.78	<.001	1.47	>.10	<1	>.10
**Arousal**	4.63 ± 0.15	4.48 ± 0.25	7.20 ± 0.68	7.65 ± 0.21	224.79	<.001	<1	>.10	2.45	>.10

Images used for the cues in Experiment 3, which were controlled for luminance, contrast, Red, Green, Blue, Jpeg, Entropy. Subjective features, such as Valence and Arousal, were also compared.

#### Data analysis

We followed the same approach for strategy analyses as in Experiments 1 and 2, but used simulations to decide on a reasonable threshold for strategy model fit, which we then preregistered. We simulated a random response pattern (the simulated participant selects outcome Y or Z for each test trial randomly) and also varied the number of test trials the simulated participant received. Strategy fit was then calculated using the procedure outlined in Experiment 1 (*strategy analyses*), to obtain a fit score for each strategy. This procedure was repeated a total of 2000 times, to obtain a mean fit score per strategy for random response patterns. This process allowed us to quantify the extent to which a random response pattern may be categorised as a real response strategy. Varying the number of test trials also helped determine the optimal number of test trials, to accurately distinguish between strategies without unnecessarily extending experiment duration. The results of this are shown in Fig. 6. Results showed that strategy fitting is biased toward fitting a simple ‘singleton’ model even if the response pattern is truly random. To minimise chances of classifying participants who responded randomly as using a “simple” strategy, we applied a threshold of 0.21, such that participants with a fit score above the threshold were classified as using ‘unidentifiable’ strategies, and replaced.

#### Statistical analysis

The Bayes Factor for the ANOVA and planned comparisons were used to evaluate the strength of evidence, with interpretation according to Lee and Wagenmakers (2014; based on Jeffreys, 1961). The analysis outcomes of associative memory accuracy and strategy followed previous experiments, except the data were now analysed in R (R version 4.2.3, http://www.r-project.org) using the package BayesFactor (Morey & Rouder, 2018). We conducted a 2-way Bayesian ANOVA (using R package BayesFactor, function *anovaBF*) on memory accuracy, with between-participant factors of Emotion (Neutral vs. Negative) and Task version (PA vs. FB). Evidence for an interaction between two factors A and B is reported by comparing a model that includes the interaction to a model that excludes it ([A*B]/[A+B]). Evidence for the main effect of A is reported by comparing a model that includes A and all its interactions with a model that excludes these ([A*B]/[B]).

After computing the best-fitting strategy for each participant, we computed the frequency of simple and complex strategies in each of the four conditions. We used *contingencyTableBF* to determine whether the frequency of using complex and simple strategies differs as the function of the factors of emotion and task versions. To do so, we computed four contingency tables: strategy-by-version for the neutral condition; strategy-by-version for the negative condition; strategy-by-emotion for FB; strategy-by-emotion for PA.

Next, strategy (complex vs. simple) was also used as an extra independent variable to assess its potential interaction with the other factors, in a three-way, between-participants Bayesian ANOVA. Given our a priori interest in the PA version, we also followed up with two, 2-way Bayesian ANOVAs, one for each Task Version: PA and FB.

To assess putative habituation to stimuli after the task, we computed several ANOVAs using the pre and posttask arousal and valence ratings. A 2-way Bayesian ANOVA with between-participant factors of Emotion (Neutral vs. Negative) and Task Version (PA vs. FB) was used to investigate pretask valence and arousal ratings, i.e., to check whether images selected for the negative (vs. neutral) condition were experienced as such. A 3-way Bayesian ANOVA with the between-participant factors of Emotion (Neutral vs. Negative), Task Version (PA vs. FB), and the within-participant factor of Image Version (Old vs. New), was used to investigate posttask valence and arousal ratings to check whether these images remained negative/neutral throughout the session. A 3-way Bayesian ANOVA with the between-participant factors of Emotion (Neutral vs. Negative), Task Version (PA vs. FB), and the within-participant factor of Image Version (Old vs. New), was also used to investigate the difference between pre and posttask valence and arousal ratings. Data were visualized using the R package ggplot2 (Wickham, 2016).

#### Exploratory statistical analysis

In addition to the preregistered statistical analyses, we conducted several exploratory analyses. First, we conducted simple effects analysis (*ttestBF*) to discern potential differences between negative and neutral stimuli for each of PA and FB conditions. We also computed learning curves for the FB condition. Using the performance from the learning phase (not test phase as in all other analyses), we computed accuracy from the first 51 trials (block 1) and next 51 trials (block 2) per emotion condition. We conducted a 2-way Bayesian ANOVA on learning accuracy, with between-participant factor of Emotion (Neutral vs. Negative) and within-participant factor Block (1 vs. 2).

### Results

After acquiring a total of 800 valid participants, we reached the maximum number of participants specified in our preregistered analyses without reaching the BF threshold for the interaction between Emotion and Task Version. The processed data and R analysis scripts are available on OSF (https://osf.io/das37/files/osfstorage?view_only=b39174160df34997b66a6b7aab668b88).

#### Manipulation check

**Fig. 7:**
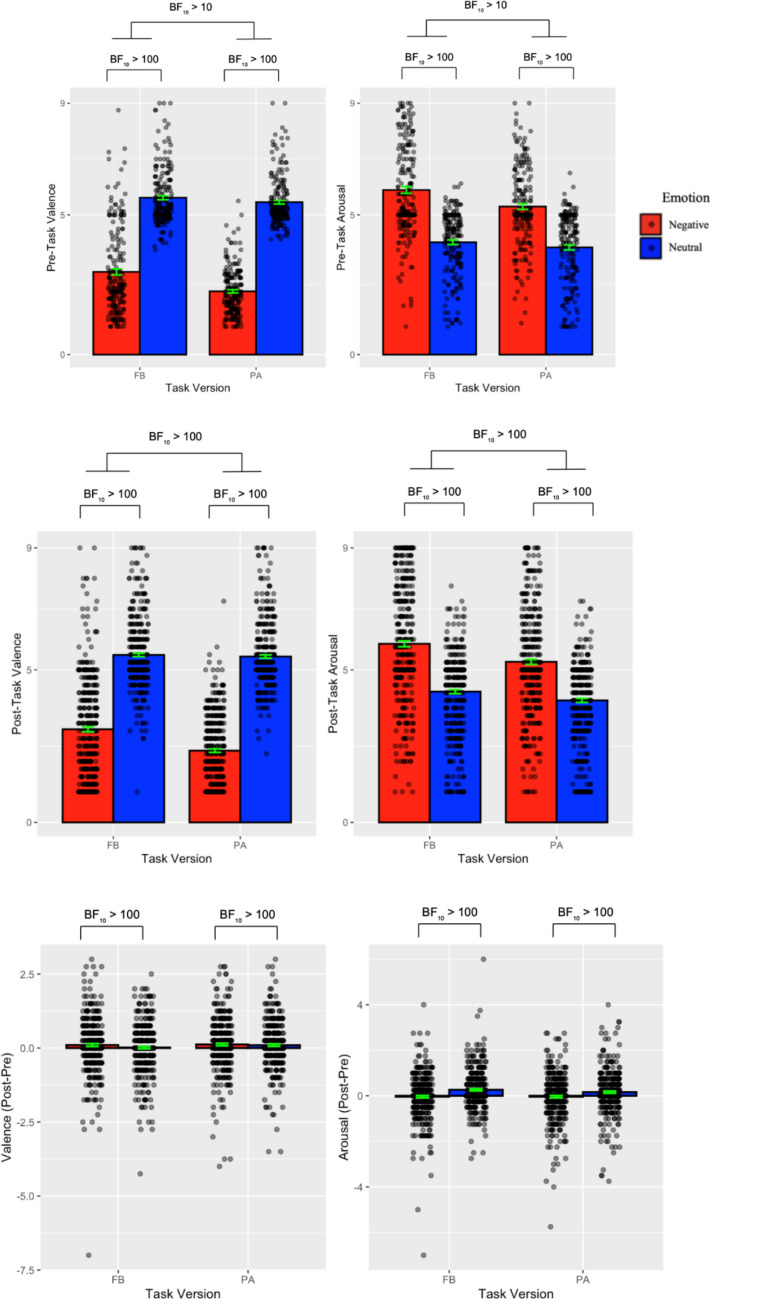
Valence and arousal ratings before and after task performance. Left: Arousal ratings; Right: Valence ratings. Top row: pretask ratings. Middle row: Posttask ratings. Bottom row: Difference in ratings from pre to posttask, with positive values indicating habituation and negative values sensitisation. BF_10_ > 10 indicates strong evidence in favour of the alternative hypothesis, BF_10_ > 100 indicates decisive evidence in favour of the alternative hypothesis.

Habituation to images was assessed in several preregistered Bayesian ANOVAs. These showed that, reassuringly, that just like in Experiment 1, negative images were rated as more negative and arousing than the neutral ones. Despite slight habituation, which we could quantify by comparing images that participants saw in the task with those they have rated initially but were not presented during the task, negative images were still rated as more negative and arousing at the end of the task (Fig. 7). The ratings also suggested that, unexpectedly, the initial task instructions participants viewed (about how to perform the FB or PA tasks, without any images) had an effect on their emotional evaluation of the images, such that following FB instructions, images were rated as less negative and more arousing compared to following PA instructions.^1^

First, we evaluated pretask arousal and valence, respectively, in two 2-way Bayesian ANOVA with between-participant factors of Emotion and Task Version. As expected, we found a significant main effect of Emotion on arousal ratings (BF_10_ = 257.98 x 10^47^) and, surprisingly, also of Task version (BF_10_ = 18.33). There was not sufficient evidence of an interaction of Emotion and Task Version (BF_10_ = 0.84) on arousal ratings. We also found a significant main effect of Emotion (BF_10_ = 54.04 x 10^167^), of Task Version (BF_10_ = 16.25) and a significant interaction of Emotion and Task Version (BF_10_ = 32.98) on valence ratings. The interaction did not qualify the effect of emotion, which was significant both in the FB (BF₁₀ = 1.19 × 10⁶⁰) and in the PA (BF₁₀ = 4.49 × 10^12^⁷) versions.

Next, we conducted two, three-way Bayesian ANOVA analyses on the posttask arousal ratings and valence ratings, adding the within-participant factor Image Condition (old vs. new) to the above statistical design. The analysis of arousal ratings revealed strong evidence for a main effect of Emotion (BF_10_ = 93.57 x 10^58^) and of Task Version (BF_10_ = 15742.83), and moderate evidence against a main effect of Image Condition (BF_10_ = 0.18). Analysis of valence ratings revealed strong evidence for a main effect of Emotion (BF_10_ = 79.07 x 10^255^) and of Task Version (BF_10_ = 136.57), and against a main effect of Image Condition (BF_10_ = 0.08). There was also strong evidence for an interaction of Task Version and Emotion (BF_10_ = 19313.72).

Finally, the difference in arousal ratings and valence ratings between post and pretask was analysed in two three-way Bayesian ANOVAs, using the same statistical design as above. The analysis of arousal ratings showed extreme evidence for a main effect of Emotion (BF_10_ = 125885.6) and moderate evidence against an effect of Task Version (BF_10_ = 0.11). There was no conclusive evidence for an effect of Image condition (BF_10_ = 0.48), nor any interactions between factors. The effect of emotion was due to a slight decrease in arousal ratings for negative images and a slight increase for neutral images. The same analysis on valence ratings showed extreme evidence for a main effect of Emotion (BF_10_ = 4294.12) and for Image condition (BF_10_ = 179.68), and moderate evidence against an effect of Task Version (BF_10_ = 0.12). There was also moderate evidence against any interaction of factors (BF_10_ < 0.16). The effect of emotion was due to the negative images being rated as slightly less negative after the task. The effect of Image condition was due to the old images (those viewed during the task) also being rated as slightly less negative.

#### Associative memory performance

As in Experiment 2, participants performed better in the FB version of the task compared to the PA version, and in the FB version, they performed slightly better in the neutral compared to the negative condition (Fig. 3, right). A 2-way Bayesian ANOVA on accuracy with between-participant factors of Emotion (Neutral vs. Negative) and Task Version (FB vs. PA) was conducted. The results provided extreme evidence for a main effect of Task Version (BF_10_ = 1.4 x 10^13^), due to higher accuracy in the FB than in the PA Task Condition. The data did not provide sufficient evidence for or against a main effect of Emotion (BF_10_ = 0.68), nor interaction of Task * Emotion (BF_10_ = 1.10). We conducted a nonregistered analysis of simple effects. For PA, there was moderate evidence against a difference between Emotion conditions (BF_10_ = 0.11), whereas for FB, there was strong evidence for a difference between emotion conditions (BF_10_ = 12.99).

#### Strategy use

To determine differences in strategy use, we preregistered subdivision of the 2x2 design into a series of different frequency tables to discern potential differences in strategy use between Emotion condition and Task Version. In brief, the data confirmed our second hypothesis – that in the neutral condition, participants will use complex strategies more than simple ones in the FB version, and simple more than complex ones in the PA version. The data disconfirmed the third hypothesis, that in the FB version, complex strategies would be used more frequently in the negative condition compared to the neutral one.

The comparison between neutral versions of each task and is shown in Table 8. The BF_10_ = 2054.52 suggests extreme evidence for a difference in distribution between Task Versions in the neutral condition. Confirming our second hypothesis, replicating Experiment 2 and classic WPT studies, more participants used complex strategy in the FB version, whereas more participant used simple strategies in the PA version. For completeness, we also compared the Emotion versions of each task in the same way (Table 9), but the evidence was inconclusive (BF_10_ = 0.81)

**Table 8:** Strategy use in neutral FB and PA version of the WPT

	FB – neutral	PA – neutral
Simple	78	122
Complex	122	78
Total	200	200

**Table 9:** Strategy use in emotional FB and PA version of the WPT

	FB – emotional	PA – emotional
Simple	114	133
Complex	86	67
Total	200	200

We next examined each task separately (Table 10). Our third hypothesis predicted greater use of complex strategies in the emotional condition, and we did obtain strong evidence (BF_10_ = 82.27) for a difference in strategy use, but in the opposite direction than predicted. Replicating Experiment 1, the data showed that more participants in the neutral version used complex strategies compared with the emotional version. For completeness, we also compared the neutral and negative version of the PA version of the task (Table 11), but the evidence was inconclusive (BF_10_ = 0.23).

**Table 10:** Strategy use neutral and emotional FB version of the task

	FB – neutral	FB – emotional
Simple	78	114
Complex	122	86
Total	200	200

**Table 11:** Strategy use neutral and emotional PA version of the task

	PA – neutral	PA – emotional
Simple	122	133
Complex	78	67
Total	200	200

#### Strategy use and associative memory performance

A 2-by-2-by-2 Bayesian ANOVA on accuracy with between-participant factors of Emotion (Neutral vs. Negative), Task Version (FB vs. PA), and Strategy Use (Complex vs. Simple) was conducted. The results provided moderate evidence against the presence of a three-way interaction (BF_10_ = 0.15). Although the pattern of results was similar in each task version (Fig. 5, bottom), we report two preregistered Emotion-by-Strategy ANOVA analyses, one for each Task Version. In the FB version there was extreme evidence for an effect of Strategy (BF_10_ = 2.9 * 10^^16^) and moderate evidence against an effect of Emotion (BF_10_ = 0.30), as well as inconclusive evidence that they interacted (BF_10_ = 0.61). In the PA version, there was again extreme evidence for an effect of Strategy (BF_10_ = 3 x 10^13^), strong evidence against an effect of emotion (BF_10_ = 0.04), as well as moderate evidence against an interaction (BF_10_ = 0.29). In a nonregistered analysis, we examined the simple effects of emotion in each Task Version, separately for groups of participants who used simple or complex strategies. There was moderate evidence against a difference in performance between the emotional and neutral conditions in each analysis (PA simple, BF_10_ = 0.25; PA complex, BF_10_ = 0.21, FB simple, BF_10_ = 0.18; FB complex, BF_10_ = 0.29). These results suggest that once strategy use is considered, emotion no longer influences task performance.

#### Learning curves

**Fig. 8:**
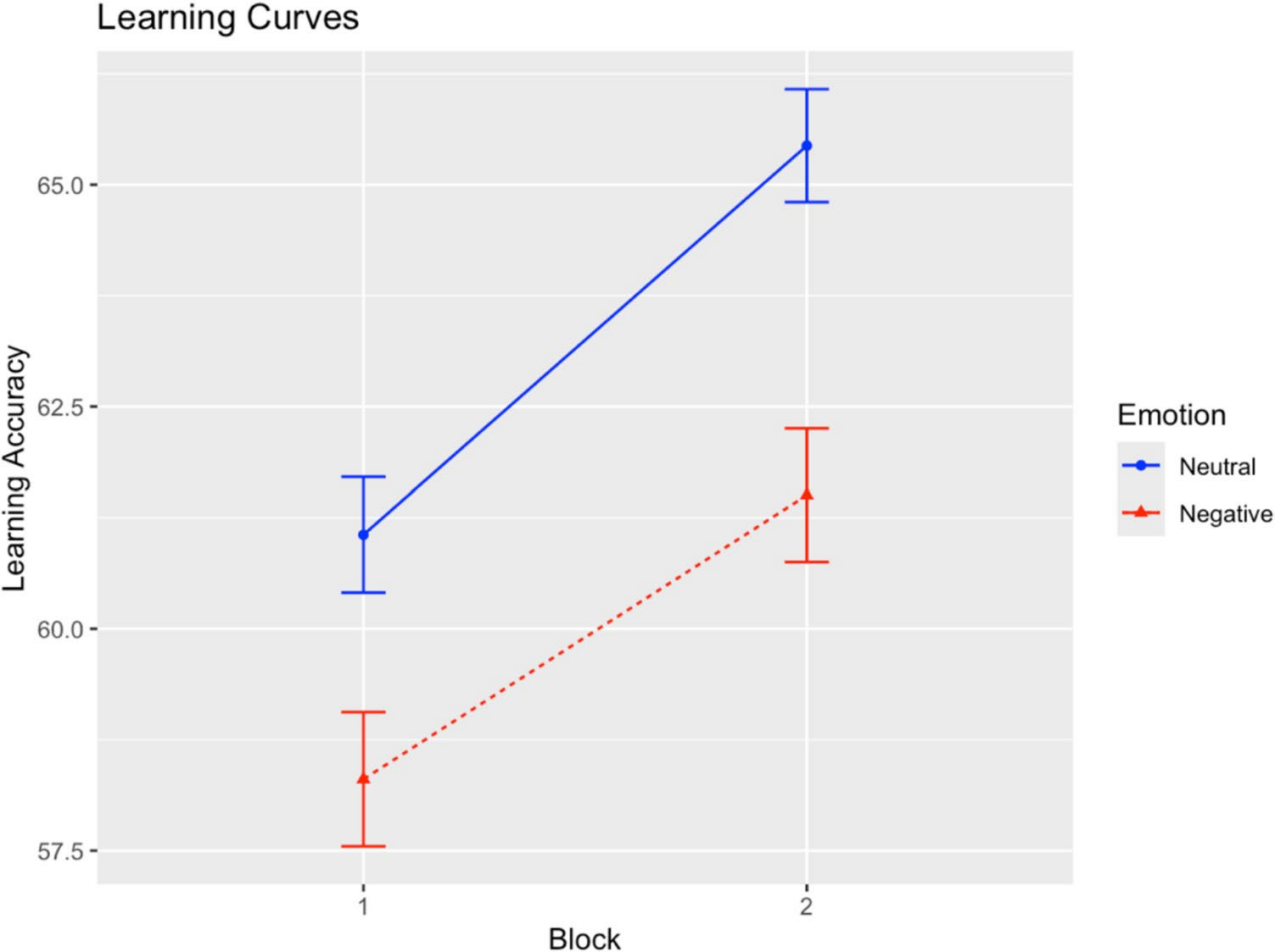
Learning Curves for FB condition, split into first 51 trials (block 1) and next 52 trials (block 2)

A 2-by-2 ANOVA was conducted on the learning performance with the between-participant factor of Emotion condition, as well as the within-participant factor Block (1 vs. 2). This analysis showed extreme evidence for a main effect of Emotion (BF_10_ = 3389) and for Block (BF_10_ = 73990.35), and moderate evidence against an interaction effect (BF_10_ = 0.163). These effects are visualised in Fig. 8.

### Discussion

We conducted a large, well-powered experiment to ascertain more conclusively whether emotional stimuli selectively impair hippocampal-dependent associative binding, operationalised through performance accuracy in the PA version and also potentially reflected in strategy use. Replicating classic WPT findings and our preregistered second hypothesis, in the neutral condition, the FB version encouraged participants to use more complex strategies, which can be at least partly supported by the striatum, while the PA version encouraged the use of simple strategies, which may be more dependent on the hippocampus.

Replicating the results of the previous two experiments, the results of Experiment 3 did not support our key hypothesis that negative emotion will decrease accuracy in the PA compared with the FB version. There was no conclusive evidence for or against an interaction between emotion and task version, despite our considerable statistical power, with *N* = 200 per group. One possibility is that there is a true difference in accuracy, but it has an even smaller effect size than what we assumed in our power simulations (Cohen’s f^2^ = 0.02). Nonregistered Bayesian analyses suggested that there was moderate evidence for a null effect of emotion in the PA version and strong evidence for emotion decreasing performance in the FB version.

Critically, preregistered analysis that controlled for the individual difference of strategy use revealed evidence for a null effect of emotion. We obtained moderate evidence for a null effect of emotion both in the group that used simple strategies and in the group that used complex strategies. Taken together, we conclude that emotion does not have a detrimental influence on associative memory and that its effect is not substantially larger in the PA compared to the FB version. These results markedly contradict the central hypothesis we derived from the DRT.

The results aligned with those of Experiment 2 and confirmed our second hypothesis that in the neutral condition, the FB version encourages participants to use more complex strategies while the PA version encouraged participants to use simple strategies. Also replicating Experiment 2 but contradicting our third hypothesis, which was derived from the DRT, participants allocated to the FB condition used simple strategies more often in the negative than in the neutral condition. We discuss these findings in greater depth in the *General Discussion* section.

The far larger sample of Experiment 3 offered extreme evidence for a slight habituation in valence ratings of studied images, while no habituation was observed in arousal ratings of studied images. Additionally, posttask, all negative images—both studied and unstudied—were rated as less negative and less arousing than pretask. The effect was small, so it did not abolish the substantial differences in valence between negative and neutral evidence posttask, for which the evidence was extreme.

Unexpectedly, participants who received instructions for the FB task rated the emotional stimuli as slightly but significantly less negative and more arousing than participants who received instructions for the PA task. Because participants were allocated randomly to all conditions, and the materials were counterbalanced, the only difference between the groups was in the instructions they received. Moreover, the age and gender distributions were broadly similar across conditions. The difference is likely to be due to the instructions, because the same pattern was observed for both stimulus sets used in each of the emotional conditions, and affected both the valence and the arousal ratings. We can only speculate that expecting an active learning task may have changed the participants’ emotional response to the stimuli or their use of the rating scales. Despite this anomaly, by comparing the posttask ratings and the change in ratings from before to after the task was completed, we can be reassured that all participants kept experiencing the negative stimuli as emotionally negative and arousing throughout the task.

## General Discussion

We conducted three preregistered experiments. The last employed a large sample size of 800 to assess the impact of emotion on associative memory, with the aim of understanding whether its impact depends on the extent to which the hippocampus supports associative binding during encoding. We operationalised hippocampal-dependent associative binding by measuring memory performance in the paired associates (PA) version of the weather prediction task (WPT). Akin to traditional paired-associate learning tasks, participants in the PA version were asked to retain associations between unrelated items—picture cues and newspaper names—an ability that depends on hippocampal learning. We predicted that negative emotional arousal would render it more difficult to use the hippocampus to form associations and therefore that PA accuracy would decrease. We derived this prediction from the DRT, a theory that originated from research on PTSD but has been extended to explain laboratory findings with milder emotional stimuli. We planned to contrast the expected detrimental effect of emotion in the PA version to the FB version of the WPT, where associative binding can benefit from striatal processes, and therefore, where memory performance is likely to depend less on hippocampal learning.

Our results did not support this key hypothesis. Crucially, none of the experiments found a difference between the negative and the neutral conditions of the PA version of the WPT. Because participants can employ various strategies to succeed in probabilistic categorisation tasks (Lewandowsky et al., 2012), and because complex strategies are superior for WPT performance, we preregistered to include strategy as an individual difference variable in some of the models used in analysis. Those models, too, showed that whether participants used simple or complex strategies, negative emotional arousal did not decrease PA memory. With the caveats discussed below, we conclude that negative emotional arousal does not have a substantial detrimental effect on hippocampal-dependent associative memory.

## Individual differences in strategy use

In addition to our key hypothesis, we preregistered two additional predictions that were based on the literature, particularly on studies that used the FB version of the WPT and those that manipulated emotion and stress. Our second hypothesis was that we would replicate the literature using the WPT by observing that in the neutral conditions, the frequency of complex strategies in the FB version will be higher than in the PA version. The results supported this hypothesis. For the first two experiments, we classified strategies both with and without the possibility of unidentifiable strategies (based on a threshold for the error in model fitting). Because we observed slightly different results with and without such a threshold, we preregistered use of a threshold in Experiment 3 and therefore discuss below only the results from analyses that included the possibility of unidentifiable strategies. The predicted main effect of Task Version, with more complex strategies in FB and more simple strategies in PA, was observed in all three experiments, reaching significance in Experiments 2 and 3. In Experiment 2, fewer participants used complex strategies in the PA version, a result that partially aligns with our hypothesis, but too many used unidentifiable strategies, particularly in the PA version, potentially obscuring the effects. In Experiment 3, we preregistered to replace participants who used unidentifiable strategies. The results fully aligned with our hypothesis, with strong evidence that in the neutral condition, more participants used simple strategies and fewer used complex strategies in the PA version. Under the assumption that the FB version of the WPT is partly striatal-dependent, this finding agrees with conclusions that the striatum can support the integration of information in probabilistic category learning (Shohamy, Myers, Onlaor et al., 2004) and with Schwabe and Wolf’s (2012) argument that simple strategies may be indicative of solving the task using hippocampal processes. As such, this finding validates our implementation of this task in an online environment.

Our third hypothesis was that in the FB version participants would use complex strategies more frequently in the negative than the neutral condition, and simple strategies more frequently in the neutral than the negative condition. In Experiment 1, no relationship was found between emotion and strategies employed. In Experiment 2, the results partially aligned with our hypothesis: fewer participants in the negative condition used complex strategies and more used unidentifiable strategies. As described above, our simulations showed that the threshold used to classify which participants used which strategies in the first two experiments may have been too low. With a more conservative, preregistered threshold, the results of Experiment 3 were conclusive, and contradicted this hypothesis. Our results provide strong evidence that in the negative condition of the FB version, complex strategies were used less often, and simple strategies were used more often, suggesting that negative emotional arousal biases participants to employ simpler strategies. Below, we discuss potential reasons why these results differ from previous studies that investigated the effects of emotion and stress on the WPT.

The finding that emotion leads to the more frequent use of simple strategies contradicts previous studies (Prince et al., 2012; Schwabe & Wolf, 2012; Zerbes & Schwabe, 2021). One potential explanation for the different findings may be in the number of trials used in the different studies, as suggested by Zerbes and Schwabe (2021). Their participants were trained on the task with either moderate (100 trials) or intensive (200 trials) practice. Afterward, they either went through a stressful protocol or a nonstressful control procedure. Results showed that stress shifted those with moderate training (100 trials) to use complex strategies. However, for those with intensive training, stress did not impact their choice of strategies, likely due to stronger memory traces formed with more training. In Experiment 3, we implemented the lower trial number that was more sensitive to stress in Zerbes and Schwabe’s study, so it is unlikely that trial number explains why our results are different from theirs.

Another explanation for the divergent results regarding strategy use in the FB version is that our emotional manipulation was weaker. Several considerations render this interpretation unlikely. Throughout the three experiments, we employed highly negative and arousing images. Our stimuli included images of people injured due to car accidents and stricken with poverty, which were likely similar to the IAPS (Lang et al., 2005) images used by Steidl and colleagues (2006; 2011). While Steidl & colleagues did not provide ratings, and thus we cannot compare their images and ours directly, both their and our images seem, at face value, to be at least as negative and arousing as the images of snakes and spiders used in LaBar’s studies (Prince et al., 2012; Thomas & LaBar, 2008). Thomas and LaBar reported that, after completion, participants were asked to rate the valence and arousal of the outcomes on a 1 (negative) to 5 (positive) manikin scale. The valence ratings of their negative images had means of 1.9 to 2.3, more negative than the means of 2.8 to 3.9 for their neutral images. The difference between our negative and neutral images of approximately 2 points on a 1 to 9 rating scale is similar to the difference between their negative and neutral images. Notably, their “fearful” group, for whom these outcomes appeared more emotional, did not show an increased use of complex strategies. In fact, a correlation analysis suggested that the more fearful participants were *less* likely to use complex strategies.

Based on this observation, we argue that the best explanation for the discrepancy between our results and the previous literature is that we manipulated affective state via negative, arousing cue images, rather than negative outcome stimuli (Prince et al., 2012; Thomas & LaBar, 2008) or prelearning stress (Schwabe & Wolf, 2012; Zerbes & Schwabe, 2021). The emotional images captured attention and made it difficult to disengage (Koster et al., 2004), so some participants based their response on single images, which in the WPT would be classified as a “simple” strategy. If this interpretation is correct, a shift towards simple strategies may be observed when people are upset by what they perceive around them, while a shift towards complex strategy may be observed by endogenous negative emotions, divorced from the content of the external environment. This hypothesis could be tested in future research.

## Theoretical implications

The evidence obtained here appears to challenge predictions that we derived from a core assumption of the DRT. The DRT contends that pathological encoding of trauma disrupts hippocampal processes, and therefore, weakens the strength of associations between elements of the trauma situation. While originally confined to the aftermath of real-life trauma, the DRT has been extended to milder manipulations of emotion in laboratory experiments. For example, Brewin and Ehlers (2024) state that “Subsequent research has identified a general pattern whereby, even in healthy people, negative material induces an upregulation of the amygdala, leading to improved item memory, accompanied by a downregulation of the hippocampus such that items are bound less to their context (Bisby & Burgess, 2017; Bisby et al., 2018).” Studies that applied this logic to milder manipulations of emotion led us to predict that negative emotional arousal would have the same effect, resulting in decreased accuracy in the PA version of the WPT. Based on Schwabe and Wolf’s (2012) statement that “Stress before learning reduced the use of single-cue strategies, indications of hippocampus-based learning,” we further predicted decreased reliance on hippocampal-dependent (simple) strategies in the FB version. Instead, three experiments suggested *equivalent* performance in the neutral and negative conditions of the PA task (with Experiment 3 enrolling 400 participants in this task and obtaining moderate evidence for a null effect) and *increased* use of simple strategies in the FB version. These results imply that more caution should be exerted before extending the DRT to account for data obtained in typical emotional memory research. In the remainder of this section, we consider neurocognitive factors which may account for this pattern of findings.

The existing literature on the effect of emotion on hippocampal-dependent associative processes during encoding paints a mixed picture. In a line of evidence which is broadly consistent with the DRT, some neuroimaging studies have suggested a potential antagonistic relationship between the amygdala and hippocampus during associative memory encoding, such that negative (vs. neutral) images lead to increased amygdalar and decreased hippocampal activation, with corresponding decreases observed in associative memory performance (Berkers et al., 2016; Bisby et al., 2016). Aligning with this logic, Murray and Kensinger (2014) reported an inverse correlation between the activity of the amygdala and hippocampus during emotional associative memory encoding. However, this notion of emotional arousal leading to a disruption of hippocampal associative binding appears to be contradicted both by the present results, which consistently demonstrated a null effect of emotion on hippocampal-dependent (PA) learning within the WPT, and by an array of prior findings of either null or positive effects of emotion on associative memory (Henson et al., 2016; Luck et al., 2014; Madan et al., 2020; Mickley Steinmetz et al., 2016; Sharot & Yonelinas, 2008). Notable among these is the beneficial effect of emotion on associative binding identified by Luck et al. (2014), who found no differences in hippocampal activity between emotional and neutral image encoding, despite significantly higher amygdalar activation in the former. Thus, a basic disruptive account, in which antagonistic interactions between the amygdala and hippocampus produce impaired associative binding, is not consistent with the present results nor with the broader literature.

A crucial factor that may provide a more comprehensive account of the effect of emotion on associative memory is its impact on attentional processes, given that these processes dictate which stimuli are amenable to integration at the point of encoding. Based on this reasoning, Bogdan et al. (2024) re-examined this question using an associative memory paradigm in which background and foreground stimuli were manipulated, aiming to assess how negative (vs. neutral) foreground stimuli impacted associative memory of neutral background content, while accounting for differences in the allocation of attention using eye-tracking. Prior to including eye-tracking data in their analyses, Bogdan et al. (2024) observed reduced memory of background stimuli which were coupled with an emotional (vs. neutral) foreground stimulus, replicating prior findings of emotion impairing associative memory. Importantly, eye-tracking data suggested that this impairment may have been due to emotional foreground stimuli biasing attention, thus reducing the scope for effective encoding of the background. Crucially, when these attentional differences were accounted for by incorporating the eye-tracking data into statistical analyses of associative memory, the initial impairing effect of emotion was reversed, such that associative memory of the background as a function of attention was improved by emotional foreground stimuli. Similarly, in a subsequent experiment in which participants were explicitly instructed to focus on the background, this enhancing (rather than impairing) effect of emotion on associative memory was replicated. At a neural level, the enhancing effect was linked to increased hippocampal and amygdalar activation, as well as enhanced functional connectivity between these structures and surrounding MTL regions. These neuroimaging findings thus reveal a synergistic interaction between emotional arousal (and corresponding amygdalar activity) and associative memory formation (and corresponding hippocampal activity)—once differential attentional processes were accounted for.

Moreover, these findings speak to the potential for studies which do not account for these attentional processes to potentially detect reduced hippocampal activation during negative associative memory formation, due to narrowed input at the point of encoding, rather than a reduced capacity for hippocampal binding. Narrowed input could therefore explain why although Bisby and colleagues (2016) observed decreased hippocampal signal during associative encoding of negative, compared to neutral items, the subsequent memory effect in the hippocampus was not modulated by emotion, suggesting that emotion-dependent processes did not drive later memory success. Thus, attentional influence may act to confound integration success during the encoding of emotional and neutral pairings, leading to the conflicting behavioural and neural evidence present in the wider literature.

The advantage of the WPT is that it measures integration success implicitly, through analysis of strategies. In the present study, strategy analysis found increased use of simpler strategies in the negative condition of the FB version, indicating that in that condition, more participants focused on individual images rather than considering all images within a cue together. The finding that negative emotional arousal narrowed the focus of participants in this manner perfectly agrees with Easterbrook’s classic cue-utilization hypothesis (1959). In the WPT, focusing on single images is a suboptimal strategy because the task is designed to favour a broader focus. Consequently, FB performance was lower in the negative compared to the neutral condition. We accounted for this effect of emotion on strategy selection by including it in the statistical model and found that negative emotional arousal had no further detrimental effect on associative memory. This is notably paralleled by Bogdan and colleagues’ (2024) finding that negative emotional arousal decreased associative memory due to its impact on attention. As in the current study, once the attentional impact—captured through eye tracking measures—were taken into account in the statistical model, the detrimental effect of emotion on associative memory was abolished and even reversed.

Overall, these considerations lend support to cognitive mediation explanations of the emotional memory advantage (Talmi, 2013; Talmi et al., 2007), particularly to the Arousal-Biased Competition (ABC) theory (Mather & Sutherland, 2011). The ABC theory contends that the effect of emotion on encoding is a function of the way it impacts prioritisation. Applying this theory to associative memory, we can derive a prediction that only when participants in the negative emotion condition do not prioritize associative learning will they exhibit decreased associative memory. This prediction aligns well with the argument put forward by Dolcos & colleagues. They have argued that the discrepancy between the findings regarding the effect of emotion on relational memory depend on the particular way in which participants attend to the relationship between an emotional element of an episode and the rest of the episode during encoding, and that the effect of the emotional element on attention depends on the task set, the stimuli, and emotion regulation processes (Dolcos et al., 2022; Bogdan et al., 2024). Applying this logic to the WPT, it is possible that because participants in the FB condition can obtain some positive trial-by-trial feedback just by focusing on single images, they would prioritize the associations between *all* cues and outcomes less than in the PA condition. If this speculation is correct, ABC theory could explain why strategy influenced memory in the FB version but not the PA version, such that memory in the negative and neutral FB conditions was equated only once strategy was controlled statistically.

Taken together, our results support the argument that emotion can impair integration in a way that is detrimental to subsequent associative memory performance, unless the choice of stimuli or task set ameliorate this impact. In tasks where the impact on integration is measured sensitively and considered in the analysis of memory performance—here through WPT strategies and through eye tracking in Bogdan et al. (2024)—the impact of emotion can more directly be attributed to poor integration during encoding. Future studies of emotional associative memory should include a measure of prioritization at encoding, in addition to any memory read-outs of this effect. Such practice could help resolve some of the discrepancy between the results reported in the literature (e.g., as reviewed by Dolcos et al., 2014; Talmi & Palombo, 2024).

## Caveats

No behavioural task is process-pure, and this is also true for the WPT. Although it is agreed that the FB version of the WPT relies preferentially on striatal processes (Poldrack et al., 2001; Shohamy, Myers, Grossman et al., 2004), it is not process-pure (Hopkins et al., 2004; Lagnado et al., 2006; Newell et al., 2007; Sučević & Schapiro, 2023). While hippocampal-dependent relational binding is necessary for encoding of associations between cues and outcomes in the PA version of the WPT, and the PA version is fashioned after traditional paired-associates learning tasks, the FB task could also benefit from hippocampal processes. For example, even though higher working memory load and elaborative processing decreased performance in the PA but not FB version of the task (Li et al., 2016), Lagnado and colleagues (2006), who asked participants specific questions about the probability of each outcome for each cue, found that participants had accurate knowledge, suggesting that they may employ declarative memory to solve the FB version. Prince et al. (2012) reported that in the FB version, fearful outcomes resulted in a reduced activity of the caudate nucleus and several MTL structures, such as the amygdala, as well as a decreased striatal-MTL connectivity. Importantly, these considerations do not detract from our findings that in PA, a task more sensitive to hippocampal contribution, negative emotional arousal did not decrease memory performance. Moreover, when we controlled for individual differences in strategy preferences, there was evidence for a null effect of emotion in both task versions. Thus, while there are grounds to question the process-purity of the FB version of the WPT, our use of a PA version and of analyses controlling for strategies used, mean that our results nonetheless provide compelling support for a null effect of emotion on associative memory.

Of course, the superficial alterations made to the WPT canonical paradigm in the present research (i.e., use of emotional images as cues rather than tarot cards, newspapers as outcomes rather than weather, and photographer rather than weatherman role) could conceivably have impacted the process-purity of the two task versions and the neural bases of response strategies therein. Nonetheless, the PA version should in principle have recruited the hippocampus to a similar degree to paired-associates tasks with singular encoding trials (such as those used in Bisby et al., 2016), given that the hippocampus has been implicated in learning flexible associations between several stimuli (Bunsey & Eicehnbaum, 1996), as well as in associative learning in the context of repeated encoding trials (Lavenex et al., 2006; Law et al., 2005). It is therefore reasonable to interpret accuracy in the PA version of the WPT in this study as indicative of the fidelity of hippocampal-dependent associative binding.

It is noteworthy that we observed a difference in accuracy between the FB and PA versions, as this contrasts with previous literature (Newell et al., 2007; Poldrack et al., 2001). Although extensive efforts were made to match the two versions, such as asking participants to press on the outcome in the PA version even if there was only one outcome, there remains a possibility that participants disengaged more rapidly in the PA version. We speculate that this may have occurred due to the online environment, where the active trial-by-trial engagement required in the FB version may have kept participants more focused on the task than the PA version, thereby leading to a discrepancy in accuracy.

It should be acknowledged that the present WPT approach differed from most other investigations of the effect of emotion on associative memory, in that we presented the same negative images multiple times. To account for this, we quantified habituation by asking participants to rate two sets of images before and after the session, only one of which was used in the task, and compared the ratings of the two sets. As predicted, while there was a robust attenuation of ratings posttask, it was very small. There was extreme evidence that negative images were still rated as more negative and arousing than the neutral ones posttask, suggesting that the manipulation of emotion employed here endured across the WPT session. This is not surprising given findings that the emotion-dependent increase in the magnitude of the late positive potential ERP remained unchanged even through 60 presentations of the same image (Codispoti et al., 2006; Ferrari et al., 2011). That said, there is evidence for more rapid habituation of skin conductance and the amygdala BOLD signal when the same stimulus is repeated multiple times (Bradley et al., 1993; Wen et al., 2022), which leaves open the possibility that some components of the emotional response did habituate more substantially. As such, our results could be seen as presenting a boundary condition for the claim that negative emotional arousal decreases associative memory: a detrimental effect may manifest only when stimuli are encoded once.

## Conclusions

Three experiments were conducted to compare the effect of emotion on hippocampal- versus striatal-dependent memory. The WPT was chosen as a task suitable to address this, because its two versions (PA and FB) have been shown to recruit different memory systems, and their design can easily be matched. The WPT thus permits a direct comparison of differential reliance on hippocampal-dependent associative binding, offering a highly controlled, pared-down methodology to examine the effect of emotion on associative memory, and isolate hippocampal contribution to this process. This allowed us to test the predictions of DRT that negative stimuli impair hippocampal, associative encoding processes. With the caveats above, the results provide evidence against DRT, in that that emotion did not decrease accuracy to a greater extent in the PA than FB version of the WPT; indeed, it did not decrease associative memory in the PA version at all.

There is still controversy about whether DRT can be extended to experimental emotional manipulations, such as the pictorial stimuli used here (rather than true trauma). Even the validity of the best laboratory trauma analogue—the trauma film paradigm—has been questioned (Brewin & Field, 2024). Our results add to this debate, implying that more caution should be exerted before extending DRT to account for data obtained in typical emotional memory research using negative images, despite subjective confirmation of their negative valence and high arousal.

A detailed examination of the pattern of data we obtained, especially the consideration of the impact of emotion on integration during encoding and review of findings from recent research, suggest that the effect of laboratory-induced negative emotional arousal on associative memory in healthy participants is most likely due to its impact on prioritisation during encoding, and its impact on attention. Subsequent associative memory does not suffer from the presence of negative emotional arousal when the set-up encourages people to associate emotional items and their context.

## Data Availability

Data and materials for the reported experiments are available here: https://osf.io/das37/files/osfstorage?view_only=b39174160df34997b66a6b7aab668b88.

## Footnotes

1 Reinspection of the arousal and valence rating scales utilised in this experiment revealed a technical error in these scales (slight misalignment of the “Neutral” marker situated above 4 on the 1-9 scales), which occurred during data collection for the final 386 participants tested. Re-analysis of these data after removing the participants with misaligned scales was conducted to assess whether the pretask differences in image ratings between the PA and FB task versions was attributable to this scale misalignment issue. This analysis showed that, the same pretask effects were still observed by using only the subsample (*N* = 414) with correctly aligned rating scales.

## Code availability

Code for the reported experiments is available here: https://osf.io/das37/files/osfstorage?view_only=b39174160df34997b66a6b7aab668b88.

## Open Practices Statement

Data, materials and code are available on OSF via the following link: https://osf.io/das37/files/osfstorage?view_only=b39174160df34997b66a6b7aab668b88.

All experiments were preregistered on OSF (Experiment 1: https://osf.io/a7r8x/?view_only=66dd0c19718b43c9a76705321488832f, Experiment 2: https://osf.io/vyrtj?view_only=f539f2a6dd6345678da2945c9ce9ab93, Experiment 3: https://osf.io/hpkba?view_only=b39174160df34997b66a6b7aab668b88). All deviations from preregistration are clearly stated in the method or results sections.
